# Supplementary material for: Health system’s availability and readiness of health facilities for chronic non-communicable diseases: Evidence from the Ethiopian national surveys
Source: PLoS One. 2024 Feb 23;19(2):e0297622. doi: 10.1371/journal.pone.0297622 (PMC10890768; doi:10.1371/journal.pone.0297622)
Supplement: Attachment — Submitted filename: PONE-D-22-03523_R1 -cg.pdf [file pone.0297622.s004.pdf]

PLOS ONE

Health System’s Availability and Readiness of Health Facilities for Chronic Non-Communicable Diseases: Evidence from the Ethiopian National Surveys

--Manuscript Draft--

**Manuscript Number:**	PONE-D-22-03523R1
**Article Type:**	Research Article
**Full Title:**	Health System’s Availability and Readiness of Health Facilities for Chronic Non-Communicable Diseases: Evidence from the Ethiopian National Surveys
**Short Title:**	Health System’s Availability and Readiness of Health Facilities for Chronic Non-Communicable Diseases
**Corresponding Author:**	Sibhatu Biadgilign, MPH, MSc, PhD<br>Independent Researcher<br>Addis Ababa, ETHIOPIA
**Keywords:**	Health System's, Availability, Readiness, Services Provision, Chronic Non-Communicable Diseases
**Abstract:**	Introduction: Non-communicable diseases (NCDs) currently cause more deaths than all other causes of deaths. Cardiovascular disease, diabetes, cancer, and chronic respiratory diseases-threaten the health and economies of individuals and populations worldwide. This study aimed to assess health system’s availability and readiness of health facilities for chronic non-communicable diseases (NCDs) and describe the changes of service availability for common NCDs in Ethiopia.<br>Methods: We used data from the 2014 Ethiopia Service Provision Assessment Plus (ESPA +) and 2016 and 2018 Service Availability and Readiness Assessment (SARA) surveys, which were cross-sectional health facilities-based studies. A total of 873 health facilities in 2014, 547 in 2016, 632 in 2018 were included in the analysis. (ESPA+) and SARA surveys are conducted as a census or a nationally/sub-nationally representative sample of health facilities. Proposition of facilities that offered the service for diabetes, cardiovascular disease, chronic respiratory disease, cancer diseases, mental illness, and chronic renal diseases was calculated to measure health service availability. The health facility service readiness was measured using the mean availability of tracer items that are required to offer the service. Thus, 13 tracer items for diabetes disease, 12 for cardiovascular disease, 11 for chronic respiratory disease and 11 cervical cancer services were used.<br>Results: The services available for diagnosis and management of diabetes had not shown improvement between 2014 and 2018 with 59% in 2014, 22% in 2016, and 36% in 2018. Similarly, for cardiovascular disease, 73%, 41%, 49% in 2014, 2016, and 2018, respectively. Regarding, chronic respiratory disease; 76% in 2014, 45% in 2016 and 53 % in 2018. At the national level in 2014, the mean availability of tracer items for diabetes, cardiovascular disease, chronic respiratory disease, cancer diseases, mental illness and chronic renal diseases were 37%, 36%, 26%, 6%, 7% and 7%, respectively. In 2016, the mean availability of tracer items for diabetes, cardiovascular disease, chronic respiratory disease and cervical cancer were 53%, 41%, 27% and 72%, respectively. Similarly, in 2018, the mean availability of tracer items for diabetes, cardiovascular disease, chronic respiratory disease and cervical cancer were 48%, 42%, 27% and 51%, respectively.<br>Conclusions: The majority of the health facilities have low and gradual decrement in the availability to provide NCDs services in Ethiopia. There is a need to increase NCD service availability and readiness at primary hospitals and health centers, and private and rural health facilities where the majority of the population need the services.
**Order of Authors:**	Atkure Defar
	Girum Taye
	Della Berhanu
	Ephrem Tekle Lemango
	Abebe Bekele

Powered by Editorial Manager® and ProduXion Manager® from Aries Systems Corporation

	Kassahun Alemu
	Sibhatu Biadgilign, MPH, MSc, PhD
**Opposed Reviewers:**	
**Response to Reviewers:**	Dear Oriel Jerome Delas Alas Vida<br>PLOS ONE<br><br>Greetings of the days! Please kindly find below response for the request made during the review process. I hope this will enable to further process the manuscript.<br><br>a) Please indicate the ethical or legal reasons that have led the data to be restricted (e.g., data contain potentially identifying or sensitive patient information).<br>•There is no ethical or legal reason, but it's all about ownership. Originally, the data owner is the "National Data Management Center (NDMC)" at Ethiopian Public Health Institute (EPHI). National health and health related data have their own data governance system and shall be managing under the national data sharing policy. We have included the links to access the data. Please see below the links posted.<br>b) Include the name of the ethics committee, Institutional Review Board, or other organization that is imposing the restriction.<br>•The Ethiopian Public Health Institute (EPHI). The institute has its own data repository system at the NDMC, anyone interested to use the data sets could communicate to the center using the following link; https://rtds.ephi.gov.et/public/showdetail/94#step-1<br>c) Provide a non-author, institutional point of contact (and contact information) that is able to field data access queries. PLOS ONE's data policy requires this in the interest of maintaining long-term data accessibility.<br>•The data can be accessed using following URL- ESPA+ 2014 (https://rtds.ephi.gov.et/public/showdetail/76), SARA 2016 (https://rtds.ephi.gov.et/public/showdetail/93), SARA 2018 (https://rtds.ephi.gov.et/public/showdetail/94)<br>d) If relevant, add any data set names, variables, accession codes, URLs, DOIs, etc. that an independent researcher would need in order to request your minimal data set.<br>•The data are available upon request from the National Data Management Center (NDMC), EPHI website but are not available in public repository because the original data set is owned by NDMC) not by the authors. Anyone may contact or visit NDMC at https://rtds.ephi.gov.et/public/showdetail/94#step-1 to access the data.<br><br>With regards,
**Additional Information:**	
**Question**	**Response**
**Financial Disclosure**<br><br>Enter a financial disclosure statement that describes the sources of funding for the work included in this submission. Review the submission guidelines for detailed requirements. View published research articles from *PLOS ONE* for specific examples.<br><br>This statement is required for submission and **will appear in the published article** if the submission is accepted. Please make sure it is accurate.	The authors received no specific funding for this work.

**Unfunded studies**<br>Enter: *The author(s) received no specific funding for this work.*<br><br>**Funded studies**<br>Enter a statement with the following details:<br>• Initials of the authors who received each award<br>• Grant numbers awarded to each author<br>• The full name of each funder<br>• URL of each funder website<br>• Did the sponsors or funders play any role in the study design, data collection and analysis, decision to publish, or preparation of the manuscript?<br>• **NO** - Include this sentence at the end of your statement: *The funders had no role in study design, data collection and analysis, decision to publish, or preparation of the manuscript.*<br>• **YES** - Specify the role(s) played.<br><br>* typeset	
**Competing Interests**<br><br>Use the instructions below to enter a competing interest statement for this submission. On behalf of all authors, disclose any competing interests that could be perceived to bias this work—acknowledging all financial support and any other relevant financial or non-financial competing interests.<br><br>This statement is required for submission and **will appear in the published article** if the submission is accepted. Please make sure it is accurate and that any funding sources listed in your Funding Information later in the submission form are also declared in your Financial Disclosure statement.<br><br>View published research articles from *PLOS ONE* for specific examples.	The authors have declared that no competing interests exist.

**NO authors have competing interests**<br><br>Enter: *The authors have declared that no competing interests exist.*<br><br>**Authors with competing interests**<br><br>Enter competing interest details beginning with this statement:<br><br>*I have read the journal's policy and the authors of this manuscript have the following competing interests: [insert competing interests here]*<br><br>* typeset	
**Ethics Statement**<br><br>Enter an ethics statement for this submission. This statement is required if the study involved:<br><br>• Human participants<br>• Human specimens or tissue<br>• Vertebrate animals or cephalopods<br>• Vertebrate embryos or tissues<br>• Field research<br><br>Write "N/A" if the submission does not require an ethics statement.<br><br>General guidance is provided below. Consult the submission guidelines for detailed instructions. **Make sure that all information entered here is included in the Methods section of the manuscript.**	The current study was conducted based on a secondary analysis of existing data sets from the three-wave of data collection (2014, 2016, and 2018). Ethical clearance was obtained from the scientific Ethical Review Committee (SERC) of the Ethiopian Public Health Institute (EPHI 6.13/966). Informed verbal consent was obtained from all health facilities in-charge and health workers responsible for client services who were present at the facility. Informed consent was obtained from the participant.

Powered by Editorial Manager® and ProduXion Manager® from Aries Systems Corporation

**Format for specific study types**<br><br>**Human Subject Research (involving human participants and/or tissue)**<br>• Give the name of the institutional review board or ethics committee that approved the study<br>• Include the approval number and/or a statement indicating approval of this research<br>• Indicate the form of consent obtained (written/oral) or the reason that consent was not obtained (e.g. the data were analyzed anonymously)<br><br>**Animal Research (involving vertebrate animals, embryos or tissues)**<br>• Provide the name of the Institutional Animal Care and Use Committee (IACUC) or other relevant ethics board that reviewed the study protocol, and indicate whether they approved this research or granted a formal waiver of ethical approval<br>• Include an approval number if one was obtained<br>• If the study involved *non-human primates*, add *additional details* about animal welfare and steps taken to ameliorate suffering<br>• If anesthesia, euthanasia, or any kind of animal sacrifice is part of the study, include briefly which substances and/or methods were applied<br><br>**Field Research**<br><br>Include the following details if this study involves the collection of plant, animal, or other materials from a natural setting:<br>• Field permit number<br>• Name of the institution or relevant body that granted permission	
**Data Availability**<br><br>Authors are required to make all data underlying the findings described fully available, without restriction, and from the time of publication. PLOS allows rare exceptions to address legal and ethical concerns. See the PLOS Data Policy and FAQ for detailed information.	No - some restrictions will apply

Powered by Editorial Manager® and ProduXion Manager® from Aries Systems Corporation

A Data Availability Statement describing where the data can be found is required at submission. Your answers to this question constitute the Data Availability Statement and **will be published in the article**, if accepted.<br><br>**Important:** Stating 'data available on request from the author' is not sufficient. If your data are only available upon request, select 'No' for the first question and explain your exceptional situation in the text box.<br><br>Do the authors confirm that all data underlying the findings described in their manuscript are fully available without restriction?	
**Describe where the data may be found in full sentences. If you are copying our sample text, replace any instances of XXX with the appropriate details.**<br><br>• If the data are **held or will be held in a public repository**, include URLs, accession numbers or DOIs. If this information will only be available after acceptance, indicate this by ticking the box below. For example: *All XXX files are available from the XXX database (accession number(s) XXX, XXX.*).<br>• If the data are all contained **within the manuscript and/or Supporting Information files**, enter the following: *All relevant data are within the manuscript and its Supporting Information files.*<br>• If neither of these applies but you are able to provide **details of access elsewhere**, with or without limitations, please do so. For example:<br><br>*Data cannot be shared publicly because of [XXX]. Data are available from the XXX Institutional Data Access / Ethics Committee (contact via XXX) for researchers who meet the criteria for access to confidential data.*<br><br>*The data underlying the results presented in the study are available from (include the name of the third party*	The author of this manuscript has no right to share or put the data on a public data repository to be accessible by all who need the data, it can only be accessed through a formal request to the Ethiopian Public Health institute, National Data Management Centre.

and contact information or URL).<br>• This text is appropriate if the data are owned by a third party and authors do not have permission to share the data.<br><br>* typeset	
Additional data availability information:	

Dear Editor,

PLOS ONE Editorial Office,

Please consider the enclosed manuscript entitled **“Health System’s Availability, Readiness and Services Provision of Health Facilities for Chronic Non-Communicable Diseases: Evidence from the Ethiopian National Surveys”** for publication in journal of PLOS ONE.

It is one of the neglected areas in non-communicable disease matter addressing the country level management of diabetes, cardiovascular disease, chronic respiratory disease, cancer diseases, mental illness and chronic renal diseases and associated factor in sub-Sahara Africa set up in a body of literature. Therefore, this we help to see the extent of diabetes, cardiovascular disease, chronic respiratory disease, cancer diseases, mental illness and chronic renal diseases matter becoming a recent phenomena and situations in Africa in most low and middle-income country.

Decision and policy makers, activists, researchers use the article for program and interventional effort to maintain the necessary action to mitigate the impact it brought for the cardiovascular risk factor and advocate safeguarding for health and life. Again, it fills the gap of the lack of scientific literature that designed for programmatic effort to avert for reduction of diabetes, cardiovascular disease, chronic respiratory disease, cancer diseases, mental illness and chronic renal diseases in Africa. Many scientists especially those in Africa can get this paper in fruitful manner as it help to refer this for their future use. As the paper is coming from low-income countries, we request a full waive for publication.

The manuscript is suitable for publication in journal of PLOS ONE is that it has fast publication in record of accomplishment. The manuscript is original and submitted to the journal and comments will be incorporated based on the editorial office and reviewers thought.

Kindest regards

Sibhatu Biadgilign

Manuscript
Click here to access/download;Manuscript;21 Jan_MS Trends of Availability_ 2022.doc

Health System's Availability and Readiness of Health Facilities for Chronic Non-Communicable Diseases: Evidence from the Ethiopian National Surveys

Atkure Defar[1,2], Girum Taye Zeleke[1], Della Berhanu[1,3], Ephrem Tekle Lemango[4], Abebe Bekele[1], Kassahun Alemu[2], Sibhatu Biadgilign[5*]

Affiliations

1. Ethiopian Public Health Institute, Addis Ababa, Ethiopia
2. College of Medicine and Health Science, Institute of Public Health, Department of Epidemiology and Biostatistics, University of Gondar, Gondar, Ethiopia
3. London School of Hygiene & Tropical Medicine, London, UK
4. Maternal, Child Health and Nutrition Directorate, Ministry of Health, Sudan Street, Addis Ababa, Ethiopia.
5. Public Health Nutrition Research Consultant, P.O. Box 24414, Addis Ababa, Ethiopia

Email address:

Atkure Defar: atkuredefar@gmail.com

Girum Taye Zeleke: girumt2000@yahoo.com

Della Berhanu: della.berhanu@lshtm.ac.uk

Ephrem Tekle Lemango: ephremt.lemango@gmail.com

Abebe Bekele: abebe1277belay@gmail.com

Kassahun Alemu: kassalemu@gmail.com

Sibhatu Biadgilign: sibhatu2010@gmail.com

***Corresponding author**

Sibhatu Biadgilign, PhD, MPH, MSc

P.O.Box 24414

Addis Ababa, Ethiopia

Abstract

Introduction: Non-communicable diseases (NCDs) currently cause more deaths than all other causes of deaths. Cardiovascular disease, diabetes, cancer, and chronic respiratory diseases- threaten the health and economies of individuals and populations worldwide. The overall aim of this study is to assess health system's availability and readiness of health facilities for chronic non-communicable diseases (NCDs) and describe the changes in the availability of services for common NCDs in Ethiopia.

Methods: A cross-sectional health facilities-based study was employed and analyzed using data from the 2014 Ethiopia Service Provision Assessment Plus (ESPA +) and 2016 and 2018 Service Availability and Readiness Assessment (SARA) surveys. A total of 873 health facilities in 2014, 547 in 2016, 632 in 2018 were included in the analysis. (ESPA+) and SARA surveys are conducted as a census or a nationally/sub-nationally representative sample of health facilities.

Results: The services available for diagnosis and management of diabetes had not shown improvement between 2014 and 2018 with 59% in 2014, 22% in 2016, and 36% in 2018. Similarly for cardiovascular disease, 73%, 41%, 49% in 2014, 2016, and 2018, respectively. Regarding, chronic respiratory disease; 76% in 2014, 45% in 2016 and 53 % in 2018. At the national level in 2014, the mean availability of tracer items for diabetes, cardiovascular disease, chronic respiratory disease, cancer diseases, mental illness and chronic renal diseases were 37%, 36%, 26%, 6%, 7% and 7%, respectively. In 2016, the mean availability of tracer items for diabetes, cardiovascular disease, chronic respiratory disease and cervical cancer were 53%, 41%, 27% and 72%, respectively. Similarly, in 2018, the mean availability of tracer items for diabetes, cardiovascular disease, chronic respiratory disease and cervical cancer were 48%, 42%, 27% and 51%, respectively.

Conclusions: The majority of the health facilities have low and gradual decrement in the availability to provide NCDs services in Ethiopia. Ethiopia. There is a need to increase NCD service availability and readiness at primary hospitals and health centers, and private and rural health facilities where the majority of the population receive services.

Introduction

According to the World Health Organization (WHO) each year the deaths of 40 million people were reported from non-communicable diseases (NCDs), which is equivalent to 70% of all deaths globally [1]. In Ethiopia, NCDs cause 42% of deaths, of which 27% are premature deaths before 70 years of age. The Disability Adjusted Life Years (DALYs) increased from below 20% in 1990 to 69% in 2015 and the incidence and prevalence of NCDs are become rising [2] [3-8]. Furthermore, while the annual number of deaths due to infectious diseases is projected to decline, the total annual number of NCD deaths is projected to increase to 52 million by 2030 [9-11]. The main types of NCDs are cardiovascular diseases (such as heart attack and stroke), cancers, chronic respiratory diseases (such as chronic obstructive pulmonary disease and asthma) and diabetes [1]. Cardiovascular diseases (CVDs) account for most of the annual NCD deaths (17.7 million), followed by cancers (8.8 million), respiratory diseases (3.9 million), and diabetes (1.6 million)[1]. A study of the prevalence of raised blood glucose and diabetes in Ethiopia was 6% and raised blood pressure (SBP > 140 and/or DBP > 90 mmHg) was 15.6%, [12].The death rates of NCDs causes declined by 40% from 1990 to 2015 and also from the 680.9 per 100,000 crude all-cause mortality rate, 286.9 (95% UI: 188.1–423.0) per 100,000 people were due to NCDs[13].

NCDs threaten the progress towards the 2030 Agenda for Sustainable Development, which includes a target of reducing NCD related premature deaths by one-third. In order to attain this target, each country should prioritize and implement cost-effective policies and interventions and provide health services which tackle the rapid surges of this silent epidemic. In low and middle income countries (LMICs), several interacting factors have been associated with high burden of NCDs, including few preventative and early detection services, low availability of essential treatments, and suboptimal organization in the delivery of care[14]. The assessment of the capacity of countries' to prevent and control NCDs conducted by WHO in 2015 also showed major gaps in health system capacity in many LMICs [15]. The absence of evidence-based policies and program with inadequate response at primary care level, thus perpetuating a problem that will become worse with time unless the situation is actively addressed at a critical time [16] especially with limited data from LMICs on NCD service readiness, availability and utilization in

primary care facilities[17].

In Ethiopia, there are few studies conducted on service availability and readiness for NCDs. These studies, however, are limited as they focus on a particular or selected set of NCDs [18-20]. Other studies in the country also showed that from a total 21 public health facilities surveyed, only 38.1% had all the categories of health professionals as compared to the national standards and 85.2%, of the facilities fulfilled the criteria for basic equipment and 47.7% of the facilities did not fulfill the criteria for infection prevention supplies[21] and this is linked with the availability of essential equipment and drugs, health workers' communication, health care provided, and attitude of health workers had positive association with client satisfaction with various health programs in the health system [21-26].

In Ethiopia, health facilities lack essential logistics and major service provision bottlenecks including non-functionality of the existing district coordination body and technical committees, training gaps, staff shortage, high staff turnover resulting in work related burden, fatigue and poor motivation among service providers [27]. The overall aim of the current study was to assess the availability and readiness of services for common NCDs using Ethiopian Service Provision Assessment (ESPA+) and Service Availability and Readiness Assessment (SARA) surveys in health facilities of Ethiopia.

Methods

Data Source and Study Setting

This study used and analyzed data from the 2014 Ethiopia Service Provision Assessment Plus (ESPA +) and 2016 and 2018 Service Availability and Readiness Assessment (SARA) survey datasets. (ESPA+) and SARA surveys are conducted as census or a nationally/sub-nationally representative sample of health facilities that can guide the health sector policy and planning. The surveys were conducted using similar survey methodologies for adapting the questionnaires, training, and pre-testing and data collection. ESPA+ and SARA questionnaires contain information on general and specific service availability and readiness (e.g., types of services offered, basic amenities, equipment, medicines and commodities, diagnostics, trained staff and practice guidelines). The data were collected from all nine regions of Ethiopia namely:

Tigray, Afar, Amhara, Oromia, Somali, Benishangul-gumuz, Southern People Nations and Nationalities (SNNP), Gambella, and Harari. The survey also included two city administrations, Addis Ababa and Dire Dawa. The Ethiopian health system has primary, secondary and tertiary levels of care. Five health posts, their referral health center and primary hospital comprise the primary health care unit.[28].

Study design

A cross-sectional health facilities based study was employed in the surveys. The surveys used the SARA methodology that was developed through a joint WHO and the United States Agency for International Development (USAID) collaboration. The method considers, among other thing, the service availability mapping (SAM) tool developed by WHO, and the ESPA+ tool developed by ICF International under the USAID-funded MEASURE DHS (monitoring and evaluation to assess and use results, demographic and health surveys) project [29].

Sampling of health facilities

For ESPA+ 2014, the survey used a stratified random sampling designed to provide representative samples of: health facility types, management authorities, regions, and city administrations. A list of health facilities obtained from each regional health office was used as a sampling frame. This sampling method yielded a total of 1,327 health facilities to be surveyed, which comprised of 223 hospitals, 298 health centers and 321 health posts and 485 private clinics. The sampling method for SARA 2016 and 2018 was also a nationally representative sample stratified by health facility type and managing authority. All health facilities that were in 2016 SARA were selected for the SARA 2018 survey. In the 2016 SARA survey, 705 health facilities were included, with all hospitals (228), 165 health centers, 173 clinics, and 139 health posts. Similarly, in the 2018 SARA survey, a total of 764 health facilities were assessed, which included 303 hospitals, 164 health centers, 165 clinics and 132 health posts. The 2018 SARA survey also included new hospitals that were established after the 2016 SARA survey[29-31].

Data collection tool and procedure

An adapted version of the MEASURE DHS project ESPA+ questionnaire and World Bank service delivery indicator (SDI) were used to collect data on facility characteristics, staff contingents, service availability, referral systems, drugs and equipment, laboratory services, reporting systems, and monitoring and supervision. To obtain this information, we interviewed heads of facilities or their delegates or heads of specific sections that were believed to be knowledgeable about the service provision. Inventory and inspection of the guidelines, reports, equipment, drugs and laboratory supplies were also done.

The survey data were collected using Computer-Assisted Personal Interviewing (CAPI) using the Census and Survey Processing System (CSPro) 6.3 software. Data were transferred daily to the Ethiopian Public Health Institute server. Feedback was provided to the field team before they left the selected health facility. The Ethiopian Public Health Institute (EPHI) organized the fieldwork, monitored the data transfer system and carried out extensive supervision over the duration of the fieldwork. Regional coordinators engaged in supervision and actively solved problems faced by the field team.

Data management

The survey data were collected focusing on service readiness and availability for all NCDs in the ESPA+, including diabetes disease, cardiovascular disease, chronic respiratory disease and cancer. To calculate the readiness score, 13 tracer items for diabetes disease, 12 for cardiovascular disease, 11 for chronic respiratory disease and 11 cervical cancer services were used. The availability of the services and readiness of health facilities for diabetes, cardiovascular, and chronic respiratory diseases were investigated in the ESPA+ 2014, and the two recent (2016 and 2018) SARA surveys [29-31]. The service was considered to be available if a facility manages, diagnoses, treats or refers a patient coming to the facility for common NCDs. The facility readiness was estimated based on the availability of tracer items that are necessary to provide the service [32].

Measurement of variables

The outcome variables in this study were the "availability score" and "readiness score" of the health facilities for common NCDs. We defined facility ''availability" as the percentage of facilities in the sample that said they offered the service in question, which considered whether the diagnosis and/or management services were available. Facility "readiness" was a composite measure and was restricted to the subset of facilities that offered the service. The component "domains" that make up the readiness score included staff and training; equipment; medicines and supplies, and diagnostics. For each domain, we calculated an index as the mean score of items expressed as a percentage. A readiness score of 50 signifies that, on average, half of the facilities that offered the service had each of the requisite inputs for delivering that service. The calculated indices were compared to an agreed cutoff point of 50%. A facility index that was below 50% was considered as `not ready' to manage NCDs.

Statistical analysis

Data were cleaned and analyzed using Stata 15 (StataCorp, College Station, TX). A descriptive analysis was carried out to summarize results using percentages and proportions, which were then presented in the form of tables and graphs. The analysis for availability and readiness has been done considering the WHO service availably and readiness estimation method [1]. In this study, Fisher's exact test was used to assess the difference in the mean availability of tracer items for various NCDs and across survey years. The analysis did not include health posts since, they do not have standards for NCDs, nor are they supposed to provide curative services for NCDs. The 'Strengthening the Reporting of Observational Studies in Epidemiology (STROBE) Statement' to write the manuscript (online supplementary file 1) was followed.

Quality assurance

Quality assurance involved multiple steps along a continuum of training, data collection in the field, and data processing at the central level. Quality assurance began with the recruitment of data collectors and team leaders with a health background. Team leaders played a critical role in the correct completion of the data collection tools as they reviewed all the questionnaires. The regional coordinators visited and communicated with teams regularly to provide support

and help when difficulties arose at individual facilities. The central coordinators at EPHI and members of the Technical Working Group supervised the teams in the field to ensure consistency and quality. When needed, the central data managers telephoned facilities for clarification and to ensure quality.

Ethical consideration

The current study was conducted based on a secondary analysis of existing data sets from the three-waves of data collection (2014, 2016, and 2018). Ethical clearance was obtained from the Scientific Ethical Review Committee (SERC) of the EPHI with reference number of EPHI 6.13/966. Informed verbal consent was obtained from all health facilities' in-charge and sometimes health workers responsible for client services who were present at the facility.

Patient and public involvement

Patients or the public were not involved in the design, or conduct, or reporting, or dissemination plans of this research.

Result

Characteristics of health facilities

In this comprehensive study, a total of 873 health facilities in 2014, 547 in 2016, and 632 in 2018 were included in the analysis. The results are stratified by facility type, managing authority, region, and residence (**Table 1**).

Availability of important components for the management of NCDs

The services available for the diagnosis and/or management of diabetes had not shown improvement between the periods of 2014 to 2018. The availability for diagnosis and/or management services for diabetes was 59%, 22%, and 36% in 2014, 2016, and 2018, respectively. Similarly, the availability for diagnosis and/or management services for cardiovascular disease was 73%, 41%, 49% in 2014, 2016, and 2018, respectively and 76% in 2014, 45% in 2016 and 53% in 2018 for chronic respiratory disease. Only cancer diseases, mental illness and chronic renal diseases service were diagnosed and managed as 23%, 32%

and 59% for the survey year of 2014 (**Table 1).** There were regional differences across the availability of health facilities offering the services for the management of NCDs. Mostly, the availability of essential guidelines, trained human power and materials and equipment for the management of NCDs services varied across the survey years and regions. Detail description is illustrated in **Table 1.**

Health facility readiness for the management of NCDs

Service readiness was measured generally by the availability of selected tracer items. In all categories of diseases, the readiness was checked by the availability of guideline and at least 1 trained staff as necessary tracer items to provide the service. Additionally, the analysis considered the availability items listed in **Table 2**. The general service readiness for cancer disease, mental illness and chronic renal disease were measured based on the availability of guideline and at least 1 trained staff as a common tracer items.

Service readiness is estimated among those who offered the services and was made by assessing the four domains: staff and guidelines, equipment, diagnostics, and medicines and Commodities. The bar graph shows the trend on overall readiness using the availability of tracer items for three of the selected NCDs (diabetes, cardiovascular, cervical cancer and chronic respiratory disease), which showed an improvement across different periods. We used 12 tracer items that are necessary to provide diabetes service in 2014, whereas 13 tracer items were used for both 2016 and 2018 surveys. On average, for each survey years, facilities had 4, 6, and 6 tracer items with overall readiness score of 37%, 53%, and 48%, respectively. This showed improvement over the period between 2014 and 2016. The overall readiness for cardiovascular diseases (of the total 12) did not show improvement over time, 36%, 41%, and 42% during, 2014 (10 tracer items), 2016 (of 12 tracer items), and 2018 (12 tracer items), respectively (**Figures 1**).

Of the 11 tracer items that are necessary to provide diabetes service, on average facilities had 3 tracer items with an overall readiness score of 26%. There were no changes over time 2016 (27%) and 2018 (27%), with the mean tracer item availability of 3 out of 11 items. At the national level, the mean availability of tracer items for diabetes, cardiovascular disease, chronic

respiratory disease, cancer diseases, mental illness, and chronic renal diseases were 37%, 36%, 26%, 6%, 7% and 7%, respectively in 2014. In 2016, the mean availability of tracer items for diabetes, cardiovascular disease, chronic respiratory disease and cervical cancer were 53%, 41%, 27% and 72%, respectively. Similarly, the mean availability of tracer items for diabetes, cardiovascular disease, chronic respiratory disease, and cervical cancer were 48%, 42%, 27% and 51%, respectively in 2018 **(Table 2).**

Changes in preparedness for common non-communicable diseases

Table 3 shows the differences in the mean availability of tracer item in percent for NCDs service availability between assessment years at the national level. In this study, there was an improvement from 2014 to 2016 in terms of the availability of tracer items with 16% for diabetes (statistically significant), 5% cardiovascular disease, and 1% chronic respiratory disease. However, from 2016 to 2018 survey year, there is a decrease for diabetes with 5% and 21% for cervical cancer (statistically significant) and showed almost no change for cardiovascular and chronic respiratory disease (**See table 3).**

Discussion

The study highlighted and revealed that major aspects of addressing service availability and readiness and readiness of health facilities for common NCDs services are compromised in the country across various periods. The study provides a comprehensive and in-depth analysis of availability and readiness of health facilities to provide services for NCDs in Ethiopia. The availability for diagnosis and/or management services for diabetes was 59%, 22%, and 36% in 2014, 2016, and 2018, respectively and also for cardiovascular disease, 73%, 41%, and 49 % in 2014, 2016, and 2018, for chronic respiratory disease; 76% in 2014, 45% in 2016 and 53 % in 2018 respectively. Similar finding has been documented in South Africa, which identified that the need to improve availability of NCD-trained nurses, functional equipment, and medication [33]. In Ugandan study, none of the facilities included met the WHO package of essential non-communicable (PEN) standards for essential tools and medicines to implement effective NCD interventions [34].

Studies in Ethiopia documented that the overall prevalence of chronic NCDs was 8.9% and specific prevalence were 0.5% for diabetes mellitus (DM), 2.6% for hypertension, 3.0% for cardiovascular diseases, 1.5% for asthma and 2.7% for mental illness in Gilgel Gibe Field Research Center[35] and national cardiovascular disease prevalence of 15%, cancer and chronic obstructive pulmonary disease prevalence of 4% each, and diabetes mellitus prevalence of 2%[36]. Health facilities included in this study were not well equipped to provide services for, diabetes, cardiovascular disease, and chronic respiratory disease. The trend was similar across the survey years. Diabetes had the lowest service availability, but the highest readiness score to provide service among the common NCDs. The availability of guidelines and at least one trained staff is minimal to deliver the management of diabetes, cardiovascular diseases, cervical cancer, and chronic respiratory disease services. Similarly, in a study conducted in Uganda, less than a half (46%) of the health facilities had hypertension guidelines with significant differences observed across health facility levels [37] which is one of the core aspects of the management of the chronic diseases. Similarly, a study done in Tanzania showed that in many health facilities, guidelines, diagnostic equipment, and first-line drug therapy for the primary care of NCDs were inadequate, and management, training, and reporting systems were weak [16]. Likewise, in the recent Tanzanian national survey, out of 725 health facilities involved in the study, only 28% of the assessed facilities were considered prepared for the outpatient primary care of hypertension. About 9% and 42% of the assessed facilities reported to have at least one trained staff and guidelines for hypertension, respectively [38]. In a Ghanaian study, the findings highlight the unavailability of essential medicines required for adequate management of NCDs reflected in the limited capacity at the various levels of care [39]. More importantly, there are challenges for healthcare delivery system: first the double burden of communicable diseases and non-communicable diseases such as diabetes and cardiovascular disease; the burden that chronic conditions impose on health systems and multiple chronic conditions and lastly burden on the primary healthcare [40]. According to the WHO recommendations, training of the health workforce and strengthening the capacity of the health systems, particularly at primary care level and policy options to address the prevention and control of NCDs are all priorities [41].

From our findings, trained human resources were inadequate for addressing and managing NCDs in Ethiopia. Consistent result was documented in Tanzanian health facilities for outpatient primary care study, in which trained health workers for NCDs were inadequate, and management, training, and reporting systems were weak. Only one of the 24 health facilities surveyed reported any training in the past year for hypertension or diabetes [16]. According to the national survey in Tanzania, about 26% and 13% of health facilities had guidelines and trained staff for NCDs, respectively [42]. Coupled with this, in a Ghanaian study, inadequate staffing was a major impediment for the capacity of primary healthcare facilities to serve NCD-related health needs [39]. The capacity of the primary care system in Vietnam was inadequate to serve the NCD-related health needs of the population [43]. Similarly, a study documented in Ethiopia showed that encouraging actions and results on NCDs in terms of political commitment, however, there is a gap as shown by the limited availability of resources for NCDs prevention and treatment services at the primary health care level. Shortage of essential NCD drugs and diagnostic facilities and lack of treatment guidelines are major challenges[2].

This study showed that the majority of the services for chronic disease management occurred in a referral hospital, general hospital, and primary hospital, unlike the primary health care facilities. This is also a case in Uganda whereby limiting the supply of anti-hypertensive medicines to higher-level health facilities is incongruent with the provision of high quality, chronic care for persons with hypertension in lower health facilities. In the same way, lower-level health facilities, where the population is expected to receive primary health care, should be expected to stock essential medicines for treating non-communicable diseases[44]. A similar finding is also documented in Zambia where nearly all primary health facilities studied did not meet the minimum threshold to manage NCDs [45] according to the WHO recommendations [41].

Our study has some limitations. Firstly, the sampling frames used in the surveys is limited to health facilities included in the country master health facility list and some other health facilities especially those recently constructed might not be included. Secondly, the cross-sectional nature of the study limits its value to explain the relative importance of some of the factors to be considered. In the same time, such kind of surveys conducted in some cases, some of the variables like hospitals, focusing more governmental and urban health facilities seems

oversampled. Thirdly, other concern might be regional variations in the availability and management of NCDs across the regions of the country. Lastly, as with the nature of the data, p-values estimation should be regarded as exploratory rather than for testing hypothesis.

Despite the above limitations, our study has several strengths. Firstly, this study undertook in a representative sample of health facilities as it is collected from health facilities in the country, so it might be generalisable to all facilities in the country. Secondly, the survey tools were using standardized WHO-recommended and validated data collection instrument, training and pre-testing of the tools was rigours in nature, the involvement of policymakers and health programme managers, approval makes for endorsement of the finding of the study for improving quality and management of NCDs carried out in the health facilities. In addition to this, it is a nationally representative sample and wealth of data to overlook the temporal pattern across the years were taken in which the result demonstrates the real situation of the health facilities regarding the health system's availability and readiness of the services for NCDs in Ethiopia.

The policy implications of the findings is that the temporal trend identified for the common non-communicable disease service availability observed from 2014 to 2018 has important implications for deciding the priority of the health care program and scaling up interventions for NCDs in Ethiopia and other developing countries. Health system policy should given due attention to NCDs service availability. In addition, from each specific NCDS, the government has to focus the attention towards prioritizing the each specific disease in descending order for chronic respiratory disease, cardiovascular disease and diabetes while providing and expanding the NCDs service provision in government/public health facilities as well as focusing on rural area and primary health care service where the majority of the community members served for other service outlets. Similarly, it will add a value on the existing NCDs prevention strategies that would be strengthened through updating the priority disease that need further attention and implication.

Conclusions

The majority of the health facilities have low and gradual decrement in the availability to provide NCDs services in Ethiopia. However, there is stability and incremental on readiness

score across the type of NCDs and importantly, more attention is needed on facility readiness to provide cervical cancer, mental illness, and chronic renal diseases services. There is a need to increase NCD service availability and readiness at primary hospitals and health centers, and private and rural health facilities where the majority of the population receive services. Basic diagnostic equipment and medication also need to be made available. Management of NCDs can also be improved by providing continuous training for primary health care workers. Opportunities for providing integrated care for NCDs should receive strong consideration, allowing for efficient use of the available resources at primary health care in low-income countries such as Ethiopia.

Declarations

Ethics approval and consent to participate

The current study was conducted based on a secondary analysis of existing data sets from the three-wave of data collection (2014, 2016, and 2018). Ethical clearance was obtained from the scientific Ethical Review Committee (SERC) of the Ethiopian Public Health Institute (EPHI 6.13/966). Informed verbal consent was obtained from all health facilities in-charge and sometimes health workers responsible for client services who were present at the facility. Informed consent was obtained from the participant.

Consent for publication

Not applicable.

Availability of data and materials

Data will be available from the corresponding author upon reasonable request

Competing Interests

The Authors declare that they don't have any competing interests.

Authors' Contributions

Conceptualization, AD, GT , BD, ET, AB, KA and SB; Data curation, Atkure Defar, Girum Taye Zeleke and Kassahun Alemu ; Formal analysis, Atkure Defar, Girum Taye Zeleke, Della Berhanu,

Kassahun Alemu and Sibhatu Biadgilign; Investigation, Atkure Defar, Girum Taye Zeleke, Della Berhanu, Ephrem Tekle Lemango, Abebe Bekele and Sibhatu Biadgilign; Methodology, Atkure Defar, Girum Taye Zeleke, Della Berhanu, Ephrem Tekle Lemango, Abebe Bekele, Kassahun Alemu and Sibhatu Biadgilign; Project administration, Atkure Defar; Resources, Atkure Defar and Sibhatu Biadgilign; Supervision, Della Berhanu, Ephrem Tekle Lemango , Abebe Bekele, Kassahun Alemu and Sibhatu Biadgilign; Validation, Girum Taye Zeleke and Sibhatu Biadgilign; Visualization, Ephrem Tekle Lemango, Abebe Bekele, Kassahun Alemu and Sibhatu Biadgilign; Writing – original draft, Atkure Defar, Girum Taye Zeleke, Della Berhanu, Ephrem Tekle Lemango, Abebe Bekele, Kassahun Alemu and Sibhatu Biadgilign; Writing – review & editing, Atkure Defar, Girum Taye Zeleke, Della Berhanu, Ephrem Tekle Lemango , Abebe Bekele, Kassahun Alemu and Sibhatu Biadgilign.

Acknowledgement

We acknowledged to the Ministry of Health and the Ethiopian Public Health Institute for providing the data. As well, we are highly indebted to appreciate the contribution of study participants as well as data collectors. The surveys were undertaken by the Ethiopian Public Health Institute (EPHI), which is the research arm of the Ethiopian Ministry of Health (FMoH). They were also done in collaboration with various organizations. For 2014 ESPA+, ICF International provided technical assistance and the survey was funded by the United States Agency for International Development (USAID), World Bank, Irish Aid, WHO and UNICEF. For 2016 and 2018 surveys, technical support was provided by the World Health Organization, World Bank and Global Fund. World Health Organization provided the financial support for the survey in 2016 and The World Bank provided financial support for the 2018 surveys.

Disclaimer

The views, findings and conclusions represented in this article are those of the authors and do not necessarily represent the official views of the institutions with which they are affiliated.

Funding

The study did not receive any specific grant from any funding agency in the public, commercial or not-for-profit sectors.

Reference

1. WHO. Key facts on noncommunicable diseases. World Health Organization(WHO) (Available at https://www.who.int/news-room/fact-sheets/detail/noncommunicable-diseases ; accessed at January 30, 2019) (2018).
2. Shiferaw, F.*, et al.* Non-communicable Diseases in Ethiopia: Disease burden, gaps in health care delivery and strategic directions. *Ethiop. J. Health Dev* **32**, 00-000 (2018).
3. Demilew, Y.M. & Firew, B.S. Factors associated with noncommunicable disease among adults in Mecha district, Ethiopia: A case control study. *PloS one* **14**, e0216446 (2019).
4. Abebe, S.M.*, et al.* The prevalence of non-communicable diseases in northwest Ethiopia: survey of Dabat Health and Demographic Surveillance System. *BMJ open* **7**, e015496 (2017).
5. Prevett, M. Chronic non-communicable diseases in Ethiopia-a hidden burden. *Ethiopian journal of health sciences* **22**, 1 (2012).
6. Giday, A., Wolde, M. & Yihdego, D. Hypertension, obesity and central obesity in diabetics and non diabetics in Southern Ethiopia. *Ethiopian Journal of Health Development* **24**(2010).
7. Abebe, S.M., Berhane, Y., Worku, A. & Getachew, A. Prevalence and associated factors of hypertension: a crossectional community based study in northwest ethiopia. *PloS one* **10**, e0125210 (2015).
8. Girum, T., Mesfin, D., Bedewi, J. & Shewangizaw, M. The burden of noncommunicable diseases in Ethiopia, 2000–2016: analysis of evidence from global burden of disease study 2016 and global health estimates 2016. *International journal of chronic diseases* **2020**(2020).
9. UNDESA. World Population Prospects: The 2012 Revision, Key Findings and Advance Tables. United Nations, Department of Economic and Social Affairs Population Division(UNDESA) . Working Paper No. ESA/P/WP.227 (2013).
10. Mathers, C.D. & Loncar, D. Projections of global mortality and burden of disease from 2002 to 2030. *PLoS medicine* **3**, e442 (2006).
11. WHO. World Health Organization. Projections of mortality and causes of death, 2015 and 2030 (http://www.who.int/healthinfo/global_burden_disease/projections/en/, http://www.who.int/gho/ncd/mortality_morbidity/en/, accessed 8, January 2019) (2014).
12. EPHI. Summary Report On Ethiopia Steps Survey On Risk Factors For Chronic Noncommunicable Diseases And Prevalence Of Selected NCDs. Ethiopian Public Health Institute (EPHI) and Ministry of Health (MOH) (2016).
13. Misganaw, A.*, et al.* National mortality burden due to communicable, non-communicable, and other diseases in Ethiopia, 1990-2015: findings from the Global Burden of Disease Study 2015. *Population health metrics* **15**, 29 (2017).
14. Ali, M.K.*, et al.* Systems and capacity to address noncommunicable diseases in low- and middle-income countries. *Science translational medicine* **5**, 181cm184 (2013).
15. WHO. Assessing national capacity for the prevention and control of noncommunicable diseases: report of the 2017 global survey. Geneva: World Health Organization(WHO). Licence: CC BYNC-SA 3.0 IGO. (2018).
16. Peck, R.*, et al.* Preparedness of Tanzanian health facilities for outpatient primary care of hypertension and diabetes: a cross-sectional survey. *The Lancet. Global health* **2**, e285-292 (2014).
17. Duong, D.B., Minh, H.V., Ngo, L.H. & Ellner, A.L. Readiness, Availability and Utilization of Rural Vietnamese Health Facilities for Community Based Primary Care of Non-communicable Diseases: A CrossSectional Survey of 3 Provinces in Northern Vietnam. *International journal of health policy and management* **8**, 150-157 (2019).
18. Getachew, T.*, et al.* Service availability and readiness for major noncommunicable diseases at health facilities in Ethiopia *Ethiop. J. Health Dev* **31**, 384-390 (2017).
19. Bekele, A.*, et al.* Service availability and readiness for diabetes care at health facilities in Ethiopia. *Ethiop. J. Health Dev* **31**, 110-118 (2017).
20. Gelibo, T.*, et al.* Availability and readiness of services for cancer care at health facilities in Ethiopia: Implication for action. *Ethiop. J. Health Dev* **31**, 391-396 (2017).

21. Abazinab, S., Woldie, M. & Alaro, T. Readiness of Health Centers and Primary Hospitals for the Implementation of Proposed Health Insurance Schemes in Southwest Ethiopia. *Ethiopian journal of health sciences* **26**, 449-456 (2016).
22. Kumsa, A., Tura, G., Nigusse, A. & Kebede, G. Satisfaction with emergency obstetric and new born care services among clients using public health facilities in Jimma Zone, Oromia Regional State, Ethiopia; a cross sectional study. *BMC pregnancy and childbirth* **16**, 85 (2016).
23. Asrese, K. Quality of intrapartum care at health centers in Jabi Tehinan district, North West Ethiopia: clients' perspective. *BMC health services research* **20**, 439 (2020).
24. Deribew, A.*, et al.* Capacity of health facilities for diagnosis and treatment of HIV/AIDS in Ethiopia. *BMC health services research* **18**, 535 (2018).
25. Deribew, A.*, et al.* Health system capacity for tuberculosis care in Ethiopia: evidence from national representative survey. *International journal for quality in health care : journal of the International Society for Quality in Health Care* **32**, 306-312 (2020).
26. Fisseha, G., Berhane, Y., Worku, A. & Terefe, W. Quality of the delivery services in health facilities in Northern Ethiopia. *BMC health services research* **17**, 187 (2017).
27. Fenta, E.H.*, et al.* Landscape analysis of nutrition services at Primary Health Care Units (PHCUs) in four districts of Ethiopia. *PloS one* **15**, e0243240 (2020).
28. FMoH. Health Sector Transformation Plan: 2015/16-2019/20. Addis Ababa: Ethiopia. Federal Democratic Republic of Ethiopia Ministry of Health (FMoH) (2015).
29. EPHI. Ethiopia Service Provision Assessment Plus (ESPA+) Survey 2014. Ethiopian Public Health Institute (EPHI) and Ethiopian Ministry of Health (MOH) (2014).
30. EPHI. Ethiopia Service Availability and Readiness Assessment (SARA) 2016 Summary Report. Ethiopian Public Health Institute (EPHI) and Ministry of Health (MOH) (2017).
31. EPHI. Ethiopia Service Availability and Readiness Assessment (SARA) 2018 Final Report. Ethiopian Public Health Institute (EPHI) and Ministry of Health (MOH) (2018).
32. WHO. Service Availability and Readiness Assessment (SARA). An annual monitoring system for service delivery Reference Manual. Version 2.1 September 2013. World Health Organization (WHO) WHO/HIS/HSI/RME/2013/1 (2013).
33. Maimela, E.*, et al.* Interventions for improving management of chronic non-communicable diseases in Dikgale, a rural area in Limpopo Province, South Africa. *BMC health services research* **18**, 331 (2018).
34. Rogers, H.E., Akiteng, A.R., Mutungi, G., Ettinger, A.S. & Schwartz, J.I. Capacity of Ugandan public sector health facilities to prevent and control non-communicable diseases: an assessment based upon WHO-PEN standards. *BMC health services research* **18**, 606 (2018).
35. Muluneh, A.T.*, et al.* Population based survey of chronic non-communicable diseases at gilgel gibe field research center, southwest ethiopia. *Ethiopian journal of health sciences* **22**, 7-18 (2012).
36. WHO. Noncommunicable diseases country profiles 2011. Geneva: World Health Organization(WHO); 2011. p. 207 p. (2011).
37. Musinguzi, G.*, et al.* Capacity of Health Facilities to Manage Hypertension in Mukono and Buikwe Districts in Uganda: Challenges and Recommendations. *PloS one* **10**, e0142312 (2015).
38. Bintabara, D. & Mpondo, B.C.T. Preparedness of lower-level health facilities and the associated factors for the outpatient primary care of hypertension: Evidence from Tanzanian national survey. *PloS one* **13**, e0192942 (2018).
39. Nyarko, K.M.*, et al.* Capacity assessment of selected health care facilities for the pilot implementation of Package for Essential Non-communicable Diseases (PEN) intervention in Ghana. *The Pan African medical journal* **25**, 16 (2016).
40. Bischoff, A., Ekoe, T., Perone, N., Slama, S. & Loutan, L. Chronic disease management in Sub-Saharan Africa: whose business is it? *International journal of environmental research and public health* **6**, 2258-2270 (2009).
41. WHO. Global action plan for the prevention and control of noncommunicable diseases 2013-2020. World Health Organization (WHO). ISBN 978 92 4 150623 6 (2013).
42. Shayo, F.K. & Bintabara, D. Are Tanzanian health facilities ready to provide management of chronic respiratory diseases? An analysis of national survey for policy implications. *PloS one* **14**, e0210350 (2019).

43. Van Minh, H., Do, Y.K., Bautista, M.A. & Tuan Anh, T. Describing the primary care system capacity for the prevention and management of non-communicable diseases in rural Vietnam. *The International journal of health planning and management* **29**, e159-173 (2014).
44. Armstrong-Hough, M.*, et al.* Disparities in availability of essential medicines to treat non-communicable diseases in Uganda: A Poisson analysis using the Service Availability and Readiness Assessment. *PloS one* **13**, e0192332 (2018).
45. Mutale, W.*, et al.* Assessing capacity and readiness to manage NCDs in primary care setting: Gaps and opportunities based on adapted WHO PEN tool in Zambia. *PloS one* **13**, e0200994 (2018).

Manuscript
Click here to access/download;Manuscript;MS Trends of Availability_ Revised MS 08 July 2022.doc

Health System's Availability and Readiness of Health Facilities for Chronic Non-Communicable Diseases: Evidence from the Ethiopian National Surveys

Atkure Defar[1,2], Girum Taye Zeleke[1], Della Berhanu[1,3], Ephrem Tekle Lemango[4], Abebe Bekele[1], Kassahun Alemu[2], Sibhatu Biadgilign[5*]

Affiliations

1. Ethiopian Public Health Institute, Addis Ababa, Ethiopia
2. College of Medicine and Health Science, Institute of Public Health, Department of Epidemiology and Biostatistics, University of Gondar, Gondar, Ethiopia
3. London School of Hygiene & Tropical Medicine, London, UK
4. Maternal, Child Health and Nutrition Directorate, Ministry of Health, Sudan Street, Addis Ababa, Ethiopia.
5. Public Health Nutrition Research Consultant, P.O. Box 24414, Addis Ababa, Ethiopia

Email address:

Atkure Defar: atkuredefar@gmail.com

Girum Taye Zeleke: girumt2000@yahoo.com

Della Berhanu: della.berhanu@lshtm.ac.uk

Ephrem Tekle Lemango: ephremt.lemango@gmail.com

Abebe Bekele: abebe1277belay@gmail.com

Kassahun Alemu: kassalemu@gmail.com

Sibhatu Biadgilign: sibhatu2010@gmail.com

***Corresponding author**

Sibhatu Biadgilign, PhD, MPH, MSc

P.O.Box 24414

Addis Ababa, Ethiopia

Abstract

Introduction: Non-communicable diseases (NCDs) currently cause more deaths than all other causes of deaths. Cardiovascular disease, diabetes, cancer, and chronic respiratory diseases-threaten the health and economies of individuals and populations worldwide. This study aimed to assess health system's availability and readiness of health facilities for chronic non-communicable diseases (NCDs) and describe the changes of service availability for common NCDs in Ethiopia.

Methods: We used data from the 2014 Ethiopia Service Provision Assessment Plus (ESPA +) and 2016 and 2018 Service Availability and Readiness Assessment (SARA) surveys which were cross-sectional health facilities-based studies. A total of 873 health facilities in 2014, 547 in 2016, 632 in 2018 were included in the analysis. (ESPA+) and SARA surveys are conducted as a census or a nationally/sub-nationally representative sample of health facilities. Proposition of facilities that offered the service for diabetes, cardiovascular disease, chronic respiratory disease, cancer diseases, mental illness, and chronic renal diseases was calculated to measure health service availability. The health facility service readiness was measured using the mean availably of tracer items that are required to offer the service. Thus, 13 tracer items for diabetes disease, 12 for cardiovascular disease, 11 for chronic respiratory disease and 11 cervical cancer services were used.

Results: The services available for diagnosis and management of diabetes had not shown improvement between 2014 and 2018 with 59% in 2014, 22% in 2016, and 36% in 2018. Similarly for cardiovascular disease, 73%, 41%, 49% in 2014, 2016, and 2018, respectively. Regarding, chronic respiratory disease; 76% in 2014, 45% in 2016 and 53 % in 2018. At the national level in 2014, the mean availability of tracer items for diabetes, cardiovascular disease, chronic respiratory disease, cancer diseases, mental illness and chronic renal diseases were 37%, 36%, 26%, 6%, 7% and 7%, respectively. In 2016, the mean availability of tracer items for diabetes, cardiovascular disease, chronic respiratory disease and cervical cancer were 53%, 41%, 27% and 72%, respectively. Similarly, in 2018, the mean availability of tracer items for diabetes, cardiovascular disease, chronic respiratory disease and cervical cancer were 48%, 42%, 27% and 51%, respectively.

Conclusions: The majority of the health facilities have low and gradual decrement in the availability to provide NCDs services in Ethiopia. There is a need to increase NCD service availability and readiness at primary hospitals and health centers, and private and rural health facilities where majority of the population need the services.

Introduction

According to the World Health Organization (WHO) each year the deaths of 40 million people were reported from non-communicable diseases (NCDs), which is equivalent to 70% of all deaths globally [1]. In Ethiopia, NCDs cause 42% of deaths, of which 27% are premature deaths before 70 years of age. The Disability Adjusted Life Years (DALYs) increased from below 20% in 1990 to 69% in 2015 and the incidence and prevalence of NCDs are become rising [2] [3-8]. Furthermore, while the annual number of deaths due to infectious diseases is projected to decline, the total annual number of NCD deaths is projected to increase to 52 million by 2030 [9-11]. The main types of NCDs are cardiovascular diseases (such as heart attack and stroke), cancers, chronic respiratory diseases (such as chronic obstructive pulmonary disease and asthma) and diabetes [1]. Cardiovascular diseases (CVDs) account for most of the annual NCD deaths (17.7 million), followed by cancers (8.8 million), respiratory diseases (3.9 million), and diabetes (1.6 million)[1]. A study of the prevalence of raised blood glucose and diabetes in Ethiopia was 6% and raised blood pressure (SBP > 140 and/or DBP > 90 mmHg) was 15.6%, [12].The death rates of NCDs causes declined by 40% from 1990 to 2015 and also from the 680.9 per 100,000 crude all-cause mortality rate, 286.9 (95% UI: 188.1–423.0) per 100,000 people were due to NCDs[13].

NCDs threaten the progress towards the 2030 Agenda for Sustainable Development, which includes a target of reducing NCD related premature deaths by one-third. In order to attain this target, each country should prioritize and implement cost-effective policies and interventions and provide health services which tackle the rapid surges of this silent epidemic. In low and middle income countries (LMICs), several interacting factors have been associated with high burden of NCDs, including few preventative and early detection services, low availability of essential treatments, and suboptimal organization in the delivery of care[14]. The assessment of the capacity of countries' to prevent and control NCDs conducted by WHO in 2015 also showed major gaps in health system capacity in many LMICs [15]. The absence of evidence-based policies and program with inadequate response at primary care level, thus perpetuating a problem that will become worse with time unless the situation is actively addressed at a critical time [16] especially with limited data from LMICs on NCD service readiness, availability

and utilization in primary care facilities[17].

In Ethiopia, there are few studies conducted on service availability and readiness for NCDs. These studies, however, are limited as they focus on a particular or selected set of NCDs [18-20].

Other studies in the country also showed that from a total 21 public health facilities surveyed, only 38.1% had all the categories of health professionals as compared to the national standards and 85.2%, of the facilities fulfilled the criteria for basic equipment and 47.7% of the facilities did not fulfill the criteria for infection prevention supplies[21] and this is linked with the availability of essential equipment and drugs, health workers' communication, health care provided, and attitude of health workers had positive association with client satisfaction with various health programs in the health system [21-26].

In Ethiopia, health facilities lack essential logistics and major service provision bottlenecks including non-functionality of the existing district coordination body and technical committees, training gaps, staff shortage, high staff turnover resulting in work related burden, fatigue and poor motivation among service providers [27]. The overall aim of the current study was to assess the availability and readiness of services for common NCDs using Ethiopian Service Provision Assessment (ESPA+) and Service Availability and Readiness Assessment (SARA) surveys in health facilities of Ethiopia.

Methods

Data Source and Study Setting

This study used and analyzed data from the 2014 Ethiopia Service Provision Assessment Plus (ESPA +), and 2016 and 2018 Service Availability and Readiness Assessment (SARA) survey.(ESPA+) and SARA surveys are conducted as census or a nationally/sub-nationally representative sample of health facilities that can guide the health sector policy and planning. The surveys were conducted using similar survey methodologies for adapting the questionnaires, training, and pre-testing and data collection. ESPA+ and SARA questionnaires contain information on general and specific service availability and readiness (e.g., types of services offered, basic amenities, equipment, medicines and commodities, diagnostics, trained

staff and practice guidelines). The data were collected from all nine regions of Ethiopia namely: Tigray, Afar, Amhara, Oromia, Somali, Benishangul-gumuz, Southern People Nations and Nationalities (SNNP), Gambella, and Harari. The survey also included the two city administrations, Addis Ababa and Dire Dawa. The Ethiopian health system has primary, secondary and tertiary levels of care. Five health posts, their referral health center and primary hospital comprise the primary health care unit.[28].

Study design

A cross-sectional health facilities-based study was employed in the surveys. The surveys used the SARA methodology that was developed through a joint WHO and the United States Agency for International Development (USAID) collaboration. The method considers, among other thing, the service availability mapping (SAM) tool developed by WHO, and the ESPA+ tool developed by ICF International under the USAID-funded MEASURE DHS (monitoring and evaluation to assess and use results, demographic and health surveys) project [29].

Sampling of health facilities

For ESPA+ 2014, the survey used a stratified random sampling designed to provide representative samples of: health facility types, management authorities, regions, and city administrations. A list of health facilities obtained from each regional health office was used as a sampling frame. This sampling method yielded a total of 1,327 health facilities to be surveyed, which comprised of 223 hospitals, 298 health centers and 321 health posts and 485 private clinics. The sampling method for SARA 2016 and 2018 was also a nationally representative sample stratified by health facility type and managing authority. All health facilities that were in 2016 SARA were selected for the SARA 2018 survey. In the 2016 SARA survey, 705 health facilities were included, with all hospitals (228), 165 health centers, 173 clinics, and 139 health posts. Similarly, in the 2018 SARA survey, a total of 764 health facilities were assessed, which included 303 hospitals, 164 health centers, 165 clinics and 132 health posts. The 2018 SARA survey also included new hospitals that were established after the 2016 SARA survey[29-31].

Data collection tool and procedure

An adapted version of the MEASURE DHS project SPA questionnaire with some additional country specific questions and World Bank service delivery indicator (SDI) were used to collect data on facility characteristics, staff contingents, service availability, referral systems, drugs and equipment, laboratory services, reporting systems, and monitoring and supervision[32] . To obtain this information, we interviewed heads of facilities or their delegates or heads of specific sections that were believed to be knowledgeable about the service provision. Inventory and inspection of the guidelines, reports, equipment, drugs and laboratory supplies were also done. The survey data were collected using Computer-Assisted Personal Interviewing (CAPI) programed using the Census and Survey Processing System (CSPro) 6.3 software. Data were transferred daily to the Ethiopian Public Health Institute server. Feedback was provided to the field team before they moved to the next selected health facility. The Ethiopian Public Health Institute (EPHI) organized the fieldwork, monitored the data transfer system and carried out extensive supervision over the duration of the fieldwork. Regional coordinators engaged in supervision and actively solved problems faced by the field team.

Data management

The survey data were collected focusing on service readiness and availability for all NCDs in the ESPA+, including diabetes disease, cardiovascular disease, chronic respiratory disease and cancer. To calculate the readiness score, 13 tracer items for diabetes disease, 12 for cardiovascular disease, 11 for chronic respiratory disease and 11 cervical cancer services were used. The availability of the services and readiness of health facilities for diabetes, cardiovascular, and chronic respiratory diseases were investigated in the ESPA+ 2014, and the two recent (2016 and 2018) SARA surveys [29-31]. The service was considered to be available if a facility manages, diagnoses, treats or refers a patient coming to the facility for common NCDs. The facility readiness was estimated based on the availability of tracer items that are necessary to provide the service [33].

Measurement of variables

The outcome variables in this study were the “availability score” and “readiness score” of the health facilities for common NCDs. We defined facility ‘'availability" as the percentage of facilities in the sample that said they offered the service in question, which considered whether the diagnosis and/or management services were available. Facility "readiness" was a composite measure and was restricted to the subset of facilities that offered the service. The component "domains" that make up the readiness score included trained staff, guideline, equipment/supplies; medicines and commodities, and diagnostics capacity. For each domain, we calculated an index as the mean score of items expressed as a percentage. A readiness score of 50 signifies that, on average, half of the facilities that offered the service had each of the requisite inputs for delivering that service. The calculated indices were compared to an agreed cutoff point of 50%. A facility index that was below 50% was considered as `not ready' to manage NCDs.

Statistical analysis

Data were cleaned and analyzed using Stata 15 (StataCorp, College Station, TX). A descriptive analysis was carried out to summarize results using percentages and proportions, which were then presented in the form of tables and graphs. The analysis for availability and readiness has been done considering the WHO service availably and readiness estimation method [1]. In this study, Fisher's exact test was used to assess the difference in the mean availability of tracer items for various NCDs and across survey years. The analysis did not include health posts since, they do not have standards for NCDs, nor are they supposed to provide curative services for NCDs. The ‘Strengthening the Reporting of Observational Studies in Epidemiology (STROBE) Statement’ to write the manuscript (online supplementary file 1) was followed.

Quality assurance

Quality assurance involved multiple steps along a continuum of training, data collection in the field, and data processing at the central level. Quality assurance began with the recruitment of data collectors and team leaders with a health background. Team leaders played a critical role in the correct completion of the data collection tools as they reviewed all the questionnaires.

The regional coordinators visited and communicated with teams regularly to provide support and help when difficulties arose at individual facilities. The central coordinators at EPHI and members of the Technical Working Group supervised the teams in the field to ensure consistency and quality. When needed, the central data managers telephoned facilities for clarification and to ensure quality.

Ethics approval and consideration

The current study was conducted based on a secondary analysis of existing data sets from the three-waves of data collection (2014, 2016, and 2018). Ethical clearance was obtained from the Scientific Ethical Review Committee (SERC) of the Ethiopian Public Health Institute (EPHI) with reference number of EPHI 6.13/966. Informed verbal consent was obtained from all health facilities' in-charge and health workers responsible for client services who were present at the facility.

Result

Characteristics of health facilities

In this comprehensive study, a total of 873 health facilities in 2014, 547 in 2016, and 632 in 2018 were included in the analysis. The results are stratified by facility type, managing authority, region, and residence (**Table 1**).

Availability of important components for the management of NCDs

The services available for the diagnosis and/or management of diabetes had not shown improvement between the periods of 2014 to 2018. The availability for diagnosis and/or management services for diabetes was 59%, 22%, and 36% in 2014, 2016, and 2018, respectively. Similarly, the availability for diagnosis and/or management services for cardiovascular disease was 73%, 41%, 49% in 2014, 2016, and 2018, respectively and 76% in 2014, 45% in 2016 and 53% in 2018 for chronic respiratory disease. Mostly, the availability of essential guidelines, trained human power and materials and equipment for the management of NCDs services varied across the survey years. Detail description is illustrated in **Table 1.**

Health facility readiness for the management of NCDs

Service readiness was measured generally by the availability of selected tracer items. In all categories of diseases, the readiness was checked by the availability of guideline and at least 1 trained staff as necessary tracer items to provide the service. Additionally, the analysis considered the availability items listed in **Table 2**. The general service readiness for cancer disease, mental illness, and chronic renal disease were measured based on the availability of guideline and at least 1 trained staff as a common tracer items.

Service readiness is estimated among those who offered the services and was made by assessing the four domains: staff and guidelines, equipment, diagnostics, and medicines and Commodities. The bar graph shows the trend on overall readiness using the availability of tracer items for three of the selected NCDs (diabetes, cardiovascular, cervical cancer and chronic respiratory disease), which showed an improvement across different periods. We used 12 tracer items that are necessary to provide diabetes service in 2014, whereas 13 tracer items were used for both 2016 and 2018 surveys. On average, for each survey years, facilities had 4, 6, and 6 tracer items with overall readiness score of 37%, 53%, and 48%, respectively. This showed improvement over the period between 2014 and 2016. The overall readiness for cardiovascular diseases (of the total 12) did not show improvement over time, 36%, 41%, and 42% during, 2014 (10 tracer items), 2016 (of 12 tracer items), and 2018 (12 tracer items), respectively (**Figures 1**).

Of the 11 tracer items that are necessary to provide diabetes service, on average facilities had 3 tracer items with an overall readiness score of 26%. There were no changes over time 2016 (27%) and 2018 (27%), with the mean tracer item availability of 3 out of 11 items. At the national level, the mean availability of tracer items for diabetes, cardiovascular disease, chronic respiratory disease, cancer diseases, mental illness, and chronic renal diseases were 37%, 36%, 26%, 6%, 7% and 7%, respectively in 2014. In 2016, the mean availability of tracer items for diabetes, cardiovascular disease, chronic respiratory disease and cervical cancer were 53%, 41%, 27% and 72%, respectively. Similarly, the mean availability of tracer items for diabetes, cardiovascular disease, chronic respiratory disease, and cervical cancer were 48%, 42%, 27% and 51%, respectively in 2018 **(Table 2).**

Changes in preparedness for common non-communicable diseases

Table 3 shows the differences in the mean availability of tracer item in percent for NCDs service availability between assessment years at the national level. In this study, there was an improvement from 2014 to 2016 in terms of the availability of tracer items with 16% for diabetes (statistically significant), 5% cardiovascular disease, and 1% chronic respiratory disease. However, from 2016 to 2018 survey year, there is a decrease for diabetes with 5% and 21% for cervical cancer (statistically significant) and showed almost no change for cardiovascular and chronic respiratory disease (**See table 3).** The change in mental health and chronic renal disease service readiness were not assessed since we have only one time data for the 2014.

Discussion

The study highlighted and revealed that major aspects of addressing service availability and readiness of health facilities for common NCDs services are compromised in Ethiopia across various periods. The availability for diagnosis and/or management services for diabetes was 59%, 22%, and 36% in 2014, 2016, and 2018, respectively and also for cardiovascular disease, 73%, 41%, and 49 % in 2014, 2016, and 2018, for chronic respiratory disease; 76% in 2014, 45% in 2016 and 53 % in 2018 respectively. Similar finding has been documented in South Africa, which identified that the need to improve availability of NCD-trained nurses, functional equipment, and medication [34]. In Ugandan study, none of the facilities included met the WHO package of essential non-communicable (PEN) standards for essential tools and medicines to implement effective NCD interventions [35].

Studies in Ethiopia documented that the overall prevalence of chronic NCDs was 8.9% and specific prevalence were 0.5% for diabetes mellitus (DM), 2.6% for hypertension, 3.0% for cardiovascular diseases, 1.5% for asthma and 2.7% for mental illness in Gilgel Gibe Field Research Center[36] and national cardiovascular disease prevalence of 15%, cancer and chronic obstructive pulmonary disease prevalence of 4% each, and diabetes mellitus prevalence of 2%[37].

Health facilities included in this study were not well equipped to provide services for, diabetes, cardiovascular disease, and chronic respiratory disease. The trend was similar across the survey years. Diabetes had the lowest service availability, but the highest readiness score to provide service among the common NCDs. The availability of guidelines and at least one trained staff is minimal to deliver the management of diabetes, cardiovascular diseases, cervical cancer, and chronic respiratory disease services. According to the WHO report on the health care capacity for NCD management, 2020, evidence-based guidelines for the management of NCDs need to be developed and broadly implemented to ensure appropriate diagnosis, referral and treatment. Also reported that, globally, only 48% of countries reported having evidence-based guidelines, protocols or standards for the management of all four NCDs through a primary care approach. In contrary to our study, the report revealed that, guidelines for diabetes were most widely available (84%), followed by CVDs (77%), cancer (70%) and CRDs (64%). Correspondingly, countries were most likely to have guidelines for diabetes that included referral criteria and were utilized in at least 50% of health care facilities, with 80% of countries in upper-middle-income countries reporting having such guidelines[38] The decline in the trends of service availability and readiness could be the fact that the skilled staff turnover is high form the rural to urban areas. Also, the primary health care system might not be ready in terms of infrastructure, staffing and medicine and commodities which is supposed to provide the service which makes the denominator up in calculating readiness.

Our study is consistent with the study conducted in Uganda, less than a half (46%) of the health facilities had hypertension guidelines with significant differences observed across health facility levels [39] which is one of the core aspects of the management of the chronic diseases. Similarly, a study done in Tanzania showed that in many health facilities, guidelines, diagnostic equipment, and first-line drug therapy for the primary care of NCDs were inadequate, and management, training, and reporting systems were weak [16]. Likewise, in the recent Tanzanian national survey, out of 725 health facilities involved in the study, only 28% of the assessed facilities were considered prepared for the outpatient primary care of hypertension. About 9% and 42% of the assessed facilities reported to have at least one trained staff and guidelines for hypertension, respectively [40]. In a Ghanaian study, the findings highlight the unavailability of

essential medicines required for adequate management of NCDs reflected in the limited capacity at the various levels of care [41]. More importantly, there are challenges for healthcare delivery system: first the double burden of communicable diseases and non-communicable diseases such as diabetes and cardiovascular disease; the burden that chronic conditions impose on health systems and multiple chronic conditions and lastly burden on the primary healthcare [42]. According to the WHO recommendations, training of the health workforce and strengthening the capacity of the health systems, particularly at primary care level and policy options to address the prevention and control of NCDs are all priorities [43].

From our findings, trained human resources were inadequate for addressing and managing NCDs in Ethiopia. Consistent result was documented in Tanzanian health facilities for outpatient primary care study, in which trained health workers for NCDs were inadequate, and management, training, and reporting systems were weak. Only one of the 24 health facilities surveyed reported any training in the past year for hypertension or diabetes [16]. According to the national survey in Tanzania, about 26% and 13% of health facilities had guidelines and trained staff for NCDs, respectively [44]. Coupled with this, in a Ghanaian study, inadequate staffing was a major impediment for the capacity of primary healthcare facilities to serve NCD-related health needs [41]. The capacity of the primary care system in Vietnam was inadequate to serve the NCD-related health needs of the population [45]. Similarly, a study documented in Ethiopia showed that encouraging actions and results on NCDs in terms of political commitment, however, there is a gap as shown by the limited availability of resources for NCDs prevention and treatment services at the primary health care level. Shortage of essential NCD drugs and diagnostic facilities and lack of treatment guidelines are major challenges[2].

This study showed that the majority of the services for chronic disease management occurred in a referral hospital, general hospital, and primary hospital, unlike the primary health care facilities. Although, people with, or at risk of developing, NCDs require long-term care that is proactive, patient- centered, community-based and sustainable. Such care can be delivered equitably only through robust health systems founded on strong primary health care towards the attainment of universal health coverage[38]. This is also a case in Uganda whereby limiting

the supply of anti-hypertensive medicines to higher-level health facilities is incongruent with the provision of high quality, chronic care for persons with hypertension in lower health facilities. In the same way, lower-level health facilities, where the population is expected to receive primary health care, should be expected to stock essential medicines for treating non-communicable diseases [46]. A similar finding is also documented in Zambia where nearly all primary health facilities studied did not meet the minimum threshold to manage NCDs [47] according to the WHO recommendations [43].

Our study has some limitations. Firstly, the sampling frames used in the surveys is limited to health centers/health posts included in the country master health facility list and some other health facilities especially those recently constructed might not be included. Secondly, the cross-sectional nature of the study limits its value to explain the relative importance of some of the factors to be considered. In the same time, such kind of surveys conducted in some cases, some of the variables like hospitals, focusing more governmental and urban health facilities seems oversampled. Thirdly, other concern might be regional variations in the availability and management of NCDs across the regions of the country. Lastly, as with the nature of the data, p-values estimation should be regarded as exploratory rather than for testing hypothesis.
Despite the above limitations, our study has several strengths. Firstly, this study undertook in a representative sample of health facilities as it is collected from health facilities in the country, so it might be generalisable to all facilities in the country. Secondly, the survey tools were using standardized WHO-recommended and validated data collection instrument, training and pre-testing of the tools was rigours in nature, the involvement of policymakers and health programme managers, approval makes for endorsement of the finding of the study for improving quality and management of NCDs carried out in the health facilities. In addition to this, it is a nationally representative sample and wealth of data to overlook the temporal pattern across the years were taken in which the result demonstrates the real situation of the health facilities regarding the health system's availability and readiness of the services for NCDs in Ethiopia.
The temporal trend identified for the common non-communicable disease service availability observed from 2014 to 2018 should be used to prioritize health care programs and scaling up

interventions for NCDs in Ethiopia and other developing countries. Health system policy shall given due attention to NCDs service availability. In addition, from each specific NCDs, the government has to focus the attention towards prioritizing each specific disease in descending order for chronic respiratory disease, cardiovascular disease and diabetes while providing and expanding the NCDs service provision in government/public health facilities as well as focusing on rural area and primary health care service where the majority of the community members served for other services outlet. Similarly, it will add a value on the existing NCDs prevention strategies that would be strengthened through updating the priority disease that need further attention and implication.

Conclusions

The majority of the health facilities have low and gradual decrement in the availability to provide NCDs services in Ethiopia. However, there is stability and incremental on readiness score across the type of NCDs and importantly, more attention is needed on facility readiness to provide cervical cancer, mental illness, and chronic renal diseases services. There is a need to increase NCD service availability and readiness at the primary health care system, giving special attention to the primary hospitals and health centers, and private and rural health facilities where the majority of the population receive services. Basic diagnostic equipment and medication also need to be made available. Management of NCDs can also be improved by providing continuous training for primary health care workers. Opportunities for providing integrated care for NCDs should receive strong consideration, allowing for efficient use of the available resources at primary health care in low-income countries such as Ethiopia.

Declarations

Consent for publication

Not applicable.

Availability of data and materials

The author of this manuscript has no right to share or put the data on a public data repository to be accessible by all who need the data, it can only be accessed through a formal request to the Ethiopian Public Health institute, National Data Management Centre.

Competing Interests

The Authors declare that they don't have any competing interests.

Authors' Contributions

Conceptualization, AD, GT , BD, ET, AB, KA and SB; Data curation, Atkure Defar, Girum Taye Zeleke and Kassahun Alemu ; Formal analysis, Atkure Defar, Girum Taye Zeleke, Della Berhanu, Kassahun Alemu and Sibhatu Biadgilign; Investigation, Atkure Defar, Girum Taye Zeleke, Della Berhanu, Ephrem Tekle Lemango, Abebe Bekele and Sibhatu Biadgilign; Methodology, Atkure Defar, Girum Taye Zeleke, Della Berhanu, Ephrem Tekle Lemango, Abebe Bekele, Kassahun Alemu and Sibhatu Biadgilign; Project administration, Atkure Defar; Resources, Atkure Defar and Sibhatu Biadgilign; Supervision, Della Berhanu, Ephrem Tekle Lemango , Abebe Bekele, Kassahun Alemu and Sibhatu Biadgilign; Validation, Girum Taye Zeleke and Sibhatu Biadgilign; Visualization, Ephrem Tekle Lemango, Abebe Bekele, Kassahun Alemu and Sibhatu Biadgilign; Writing – original draft, Atkure Defar, Girum Taye Zeleke, Della Berhanu, Ephrem Tekle Lemango, Abebe Bekele, Kassahun Alemu and Sibhatu Biadgilign; Writing – review & editing, Atkure Defar, Girum Taye Zeleke, Della Berhanu, Ephrem Tekle Lemango , Abebe Bekele, Kassahun Alemu and Sibhatu Biadgilign.

Acknowledgement

We acknowledged to the Ministry of Health and the Ethiopian Public Health Institute for providing the data. As well, we are highly indebted to appreciate the contribution of study

participants as well as data collectors. The surveys were undertaken by the Ethiopian Public Health Institute (EPHI), which is the research arm of the Ethiopian Ministry of Health (FMoH). They were also done in collaboration with various organizations. We would like to thank, For ICF International provided technical assistance, the United States Agency for International Development (USAID), World Bank, Irish Aid, WHO and UNICEF for their support during the 2014 ESPA+. World Health Organization, World Bank and Global Fund for their support during the 2016 and 2018 surveys.

Disclaimer

The views, findings and conclusions represented in this article are those of the authors and do not necessarily represent the official views of the institutions with which they are affiliated.

Funding

The funders had no role in study design, data collection and analysis, decision to publish, or preparation of the manuscript.” We also would like to notify that the authors received no specific funding for this work

Table 1: Percent of health facilities offering services for non-communicable diseases, by background characteristics, Ethiopia

Background Characteristics	Diabetes			Cardiovascular diseases			Chronic respiratory diseases			Cancer			Number of Facilities		
										Cancer diseases	Cervical Cancer				
	SPA+ 2014	SARA 2016	SARA 2018	SPA+ 2014	SARA 2016	SARA 2018	SPA+ 2014	SARA 2016	SARA 2018	SPA+ 2014	SARA 2016	SARA 2018	SPA+ 2014	SARA 2016	SARA 2018
**Facility type**															
Referral Hospital	97	91	97	97	84	97	97	91	97	69	56	77	2	32	31
General Hospital	97	90	97	95	92	97	96	92	95	66	38	64	7	117	116
Primary Hospital	98	89	95	100	89	94	100	89	96	65	10	42	3	61	156
Health Center	63	16	37	82	50	66	88	55	70	28	2	6	182	165	164
Higher clinic	89	69	48	87	61	48	88	69	48	60	4	29	13	23	19
Medium clinic	96	65	81	93	71	79	93	76	82	32	1	24	37	64	74
Lower Clinic	34	5	8	49	10	5	49	11	13	3	0	0	119	85	72
**Managing authority**															
Government/Public	64	19	41	83	51	68	88	56	71	29	3	8	190	320	410
Others*	228	26	32	223	30	29	208	33	35	40	1	10	173	227	222
**Location of health facilities**															
Urban	72	34	43	78	43	53	82	45	57	28	4	14	149	431	494
Rural	49	5	25	70	38	41	72	45	47	19	0	1	214	116	138
**Total**	59	22	36	73	41	49	76	45	53	23	2	9	363	547	632

*Others: Governmental (military, prison, federal police), private for-profit and NGO (mission/faith-based, nonprofit)

Table 2: Percentage of health facilities having guideline, trained staff, and equipment, diagnostic capacity and essential medicines for non-communicable diseases by year, Ethiopia

Indicators by disease	SPA+2014	SARA 2016	SARA 2018
**Diabetes**			
Guideline**	12	16	15
At least 1 trained staff	6	10	8
Blood Pressure apparatus	93	100	96
Adult weighing scale	76	97	93
Height board/stadiometer	49	-	-
Blood glucose	40	66	66
Urine protein	56	89	78
Urine glucose	52	-	-
Metformin	11	31	20
Glibenclamide	28	31	22
Injectable insulin	9	18	17
Injectable glucose	15	62	57
Measuring Tape	-	71	69
Urine dipstick ketones	-	89	78
Gliclazide/glipizide tablet	-	4	7
**Mean availability of tracer items**	**37**	**53**	**48**
**Cardiovascular disease**			
Guideline	11	9	11
At least 1 trained staff	8	7	7
Stethoscope	97	100	97
B. Pressure apparatus	92	100	95
Adult scale	74	88	90
ACE inhibitors (Enalapril)	15	25	34
Thiazide	25	20	-
Beta-blockers (Atenolol)	6	15	17
Calcium channel blockers	26	20	30
Oxygen	9	17	13
Hydrochlorothiazide tablet	-	37	47
Metformin	-	19	16
Asprin	-	53	42
**Mean availability of tracer items**	**36**	**41**	**42**
**Chronic respiratory disease**			
Guideline	15	13	10
At least 1 trained staff	9	8	6
Stethoscope	99	100	96
Peak flow meter	1	5	4
Spacers for inhalers	1	4	6
Salbutamol inhaler or tablets	43	23	39
Beclomethasone inhaler	1	3	6
Prednisolone	44	44	47
Hydrocortisone injection	14	24	30
Injectable epinephrine	51	54	39
Oxygen	9	15	13
**Mean availability of tracer items**	**26**	**27**	**27**

**Cancer diseases**			
Guideline	7	-	-
At least 1 trained staff	4	-	-
**Mean availability of tracer items**	**6**	-	-
**Mental illness**			
Guideline	9	-	-
At least 1 trained staff	5	-	-
**Mean availability of tracer items**	**7**	-	-
**Chronic renal diseases**			
Guideline	11	-	-
At least 1 trained staff	2	-	-
**Mean availability of tracer items**	**7**	-	-
**Cervical cancer**			
Guideline	-	53	49
At least 1 trained staff	-	61	30
Speculum	-	96	85
Acetic acid	-	77	38
**Mean availability of tracer items**	-	**72**	**51**

**Guideline is referring to a national guideline that the health worker should consider while treating patients for each particular diseases according to the standard set by the federal ministry of health

Table 3: Health facilities mean availability of tracer items difference in percent for non-communicable diseases service between assessment years at national level, Ethiopia

**Non-communicable diseases**	**SPA+2014 to SARA 2016**		**SARA 2016 to 2018**	
	Difference	P-value	Difference	P-value
Diabetes	16	**< 0.001**	-5	0.0754
Cardiovascular disease	5	0.1199	1	0.7183
Chronic respiratory disease	1	0.7312	0	1.000
Cervical Cancer	*	*	-21	**< 0.001**

* Not applicable

Reference

1. WHO. Key facts on noncommunicable diseases. World Health Organization(WHO) (Available at https://www.who.int/news-room/fact-sheets/detail/noncommunicable-diseases ; accessed at January 30, 2019) 2018.
2. Shiferaw F, Letebo M, Misganaw A, Feleke Y, Gelibo T, Getachew T, et al. Non-communicable Diseases in Ethiopia: Disease burden, gaps in health care delivery and strategic directions. Ethiop J Health Dev 2018;32(3):00-000.
3. Demilew YM, Firew BS. Factors associated with noncommunicable disease among adults in Mecha district, Ethiopia: A case control study. PloS one. 2019;14(5):e0216446. Epub 2019/05/30. doi: 10.1371/journal.pone.0216446. PubMed PMID: 31141502; PubMed Central PMCID: PMCPMC6542246.
4. Abebe SM, Andargie G, Shimeka A, Alemu K, Kebede Y, Wubeshet M, et al. The prevalence of non-communicable diseases in northwest Ethiopia: survey of Dabat Health and Demographic Surveillance System. BMJ open. 2017;7(10):e015496. Epub 2017/10/25. doi: 10.1136/bmjopen-2016-015496. PubMed PMID: 29061601; PubMed Central PMCID: PMCPMC5665308.
5. Prevett M. Chronic non-communicable diseases in Ethiopia-a hidden burden. Ethiopian journal of health sciences. 2012;22(Spec Iss):1.
6. Giday A, Wolde M, Yihdego D. Hypertension, obesity and central obesity in diabetics and non diabetics in Southern Ethiopia. Ethiopian Journal of Health Development. 2010;24(2).
7. Abebe SM, Berhane Y, Worku A, Getachew A. Prevalence and associated factors of hypertension: a crossectional community based study in northwest ethiopia. PloS one. 2015;10(4):e0125210. Epub 2015/04/25. doi: 10.1371/journal.pone.0125210. PubMed PMID: 25909382; PubMed Central PMCID: PMCPMC4409323.
8. Girum T, Mesfin D, Bedewi J, Shewangizaw M. The burden of noncommunicable diseases in Ethiopia, 2000–2016: analysis of evidence from global burden of disease study 2016 and global health estimates 2016. International journal of chronic diseases. 2020;2020.
9. UNDESA. World Population Prospects: The 2012 Revision, Key Findings and Advance Tables. United Nations, Department of Economic and Social Affairs Population Division(UNDESA) . Working Paper No. ESA/P/WP.227 2013.
10. Mathers CD, Loncar D. Projections of global mortality and burden of disease from 2002 to 2030. PLoS medicine. 2006;3(11):e442. Epub 2006/11/30. doi: 10.1371/journal.pmed.0030442. PubMed PMID: 17132052; PubMed Central PMCID: PMCPMC1664601.
11. WHO. World Health Organization. Projections of mortality and causes of death, 2015 and 2030 (http://www.who.int/healthinfo/global_burden_disease/projections/en/, http://www.who.int/gho/ncd/mortality_morbidity/en/, accessed 8, January 2019) 2014.
12. EPHI. Summary Report On Ethiopia Steps Survey On Risk Factors For Chronic Noncommunicable Diseases And Prevalence Of Selected NCDs. Ethiopian Public Health Institute (EPHI) and Ministry of Health (MOH) 2016.
13. Misganaw A, Haregu TN, Deribe K, Tessema GA, Deribew A, Melaku YA, et al. National mortality burden due to communicable, non-communicable, and other diseases in Ethiopia,

1990-2015: findings from the Global Burden of Disease Study 2015. Population health metrics. 2017;15:29. Epub 2017/07/25. doi: 10.1186/s12963-017-0145-1. PubMed PMID: 28736507; PubMed Central PMCID: PMCPMC5521057.

14. Ali MK, Rabadan-Diehl C, Flanigan J, Blanchard C, Narayan KM, Engelgau M. Systems and capacity to address noncommunicable diseases in low- and middle-income countries. Science translational medicine. 2013;5(181):181cm4. Epub 2013/04/19. doi: 10.1126/scitranslmed.3005121. PubMed PMID: 23596201.

15. WHO. Assessing national capacity for the prevention and control of noncommunicable diseases: report of the 2017 global survey. Geneva: World Health Organization(WHO). Licence: CC BYNC-SA 3.0 IGO. 2018.

16. Peck R, Mghamba J, Vanobberghen F, Kavishe B, Rugarabamu V, Smeeth L, et al. Preparedness of Tanzanian health facilities for outpatient primary care of hypertension and diabetes: a cross-sectional survey. The Lancet Global health. 2014;2(5):e285-92. Epub 2014/05/13. doi: 10.1016/s2214-109x(14)70033-6. PubMed PMID: 24818084; PubMed Central PMCID: PMCPMC4013553.

17. Duong DB, Minh HV, Ngo LH, Ellner AL. Readiness, Availability and Utilization of Rural Vietnamese Health Facilities for Community Based Primary Care of Non-communicable Diseases: A CrossSectional Survey of 3 Provinces in Northern Vietnam. International journal of health policy and management. 2019;8(3):150-7. Epub 2019/04/14. doi: 10.15171/ijhpm.2018.104. PubMed PMID: 30980631; PubMed Central PMCID: PMCPMC6462192.

18. Getachew T, Bekele A, Amenu K, Defar A, Teklie H, Taye G, et al. Service availability and readiness for major noncommunicable diseases at health facilities in Ethiopia Ethiop J Health Dev 2017 31((Special Issue)):384-90.

19. Bekele A, Getachew T, Amenu K, Defar A, Teklie H, Gelibo T, et al. Service availability and readiness for diabetes care at health facilities in Ethiopia. Ethiop J Health Dev 2017;31(2):110-8.

20. Gelibo T, Getachew T, Bekele A, Defar A, Amenu K, Taddesse M, et al. Availability and readiness of services for cancer care at health facilities in Ethiopia: Implication for action. Ethiop J Health Dev. 2017;31((Special Issue)):391-6.

21. Abazinab S, Woldie M, Alaro T. Readiness of Health Centers and Primary Hospitals for the Implementation of Proposed Health Insurance Schemes in Southwest Ethiopia. Ethiopian journal of health sciences. 2016;26(5):449-56. Epub 2017/04/28. doi: 10.4314/ejhs.v26i5.6. PubMed PMID: 28446850; PubMed Central PMCID: PMCPMC5389059.

22. Kumsa A, Tura G, Nigusse A, Kebede G. Satisfaction with emergency obstetric and new born care services among clients using public health facilities in Jimma Zone, Oromia Regional State, Ethiopia; a cross sectional study. BMC pregnancy and childbirth. 2016;16:85. Epub 2016/04/27. doi: 10.1186/s12884-016-0877-0. PubMed PMID: 27113573; PubMed Central PMCID: PMCPMC4843201.

23. Asrese K. Quality of intrapartum care at health centers in Jabi Tehinan district, North West Ethiopia: clients' perspective. BMC health services research. 2020;20(1):439. Epub 2020/05/21. doi: 10.1186/s12913-020-05321-3. PubMed PMID: 32429907; PubMed Central PMCID: PMCPMC7236140.

24. Deribew A, Biadgilign S, Berhanu D, Defar A, Deribe K, Tekle E, et al. Capacity of health facilities for diagnosis and treatment of HIV/AIDS in Ethiopia. BMC health services research.

2018;18(1):535. Epub 2018/07/13. doi: 10.1186/s12913-018-3347-8. PubMed PMID: 29996821; PubMed Central PMCID: PMCPMC6042210.
25. Deribew A, Dejene T, Defar A, Berhanu D, Biadgilign S, Tekle E, et al. Health system capacity for tuberculosis care in Ethiopia: evidence from national representative survey. International journal for quality in health care : journal of the International Society for Quality in Health Care. 2020;32(5):306-12. Epub 2020/04/02. doi: 10.1093/intqhc/mzaa024. PubMed PMID: 32232364; PubMed Central PMCID: PMCPMC7299195.
26. Fisseha G, Berhane Y, Worku A, Terefe W. Quality of the delivery services in health facilities in Northern Ethiopia. BMC health services research. 2017;17(1):187. Epub 2017/03/11. doi: 10.1186/s12913-017-2125-3. PubMed PMID: 28279215; PubMed Central PMCID: PMCPMC5345168.
27. Fenta EH, Endris BS, Mengistu YG, Sadamo FE, Gelan EH, Beyene TG, et al. Landscape analysis of nutrition services at Primary Health Care Units (PHCUs) in four districts of Ethiopia. PloS one. 2020;15(12):e0243240. Epub 2020/12/04. doi: 10.1371/journal.pone.0243240. PubMed PMID: 33270764; PubMed Central PMCID: PMCPMC7714176.
28. FMoH. Health Sector Transformation Plan: 2015/16-2019/20. Addis Ababa: Ethiopia. Federal Democratic Republic of Ethiopia Ministry of Health (FMoH) 2015.
29. EPHI. Ethiopia Service Provision Assessment Plus (ESPA+) Survey 2014. Ethiopian Public Health Institute (EPHI) and Ethiopian Ministry of Health (MOH) 2014.
30. EPHI. Ethiopia Service Availability and Readiness Assessment (SARA) 2016 Summary Report. Ethiopian Public Health Institute (EPHI) and Ministry of Health (MOH) 2017
31. EPHI. Ethiopia Service Availability and Readiness Assessment (SARA) 2018 Final Report. Ethiopian Public Health Institute (EPHI) and Ministry of Health (MOH) 2018.
32. WHO) WHO. Service availability and readiness assessment (SARA): an annual monitoring system for service delivery: reference manual. No. WHO/HIS/HSI/RME/2013/1. World Health Organization World Health Organization, 2015.
33. WHO. Service Availability and Readiness Assessment (SARA). An annual monitoring system for service delivery Reference Manual. Version 2.1 September 2013. World Health Organization (WHO) WHO/HIS/HSI/RME/2013/1 2013.
34. Maimela E, Alberts M, Bastiaens H, Fraeyman J, Meulemans H, Wens J, et al. Interventions for improving management of chronic non-communicable diseases in Dikgale, a rural area in Limpopo Province, South Africa. BMC health services research. 2018;18(1):331. Epub 2018/05/08. doi: 10.1186/s12913-018-3085-y. PubMed PMID: 29728147; PubMed Central PMCID: PMCPMC5935929.
35. Rogers HE, Akiteng AR, Mutungi G, Ettinger AS, Schwartz JI. Capacity of Ugandan public sector health facilities to prevent and control non-communicable diseases: an assessment based upon WHO-PEN standards. BMC health services research. 2018;18(1):606. Epub 2018/08/08. doi: 10.1186/s12913-018-3426-x. PubMed PMID: 30081898; PubMed Central PMCID: PMCPMC6080524.
36. Muluneh AT, Haileamlak A, Tessema F, Alemseged F, Woldemichael K, Asefa M, et al. Population based survey of chronic non-communicable diseases at gilgel gibe field research center, southwest ethiopia. Ethiopian journal of health sciences. 2012;22(S):7-18. Epub 2013/01/16. PubMed PMID: 23319836; PubMed Central PMCID: PMCPMC3542738.

37. WHO. Noncommunicable diseases country profiles 2011. Geneva: World Health Organization(WHO); 2011. p. 207 p. 2011.
38. WHO. Health system capacity for noncommunicable diseases management a global snapshot 2020. World Health Organization (WHO) 23 August. . 2020.
39. Musinguzi G, Bastiaens H, Wanyenze RK, Mukose A, Van Geertruyden JP, Nuwaha F. Capacity of Health Facilities to Manage Hypertension in Mukono and Buikwe Districts in Uganda: Challenges and Recommendations. PloS one. 2015;10(11):e0142312. Epub 2015/11/13. doi: 10.1371/journal.pone.0142312. PubMed PMID: 26560131; PubMed Central PMCID: PMCPMC4641641.
40. Bintabara D, Mpondo BCT. Preparedness of lower-level health facilities and the associated factors for the outpatient primary care of hypertension: Evidence from Tanzanian national survey. PloS one. 2018;13(2):e0192942. Epub 2018/02/16. doi: 10.1371/journal.pone.0192942. PubMed PMID: 29447231; PubMed Central PMCID: PMCPMC5814020.
41. Nyarko KM, Ameme DK, Ocansey D, Commeh E, Markwei MT, Ohene SA. Capacity assessment of selected health care facilities for the pilot implementation of Package for Essential Non-communicable Diseases (PEN) intervention in Ghana. The Pan African medical journal. 2016;25(Suppl 1):16. Epub 2017/02/06. doi: 10.11604/pamj.supp.2016.25.1.6252. PubMed PMID: 28149441; PubMed Central PMCID: PMCPMC5257011.
42. Bischoff A, Ekoe T, Perone N, Slama S, Loutan L. Chronic disease management in Sub-Saharan Africa: whose business is it? International journal of environmental research and public health. 2009;6(8):2258-70. Epub 2009/09/11. doi: 10.3390/ijerph6082258. PubMed PMID: 19742159; PubMed Central PMCID: PMCPMC2738886.
43. WHO. Global action plan for the prevention and control of noncommunicable diseases 2013-2020. World Health Organization (WHO). ISBN 978 92 4 150623 6 2013.
44. Shayo FK, Bintabara D. Are Tanzanian health facilities ready to provide management of chronic respiratory diseases? An analysis of national survey for policy implications. PloS one. 2019;14(1):e0210350. Epub 2019/01/08. doi: 10.1371/journal.pone.0210350. PubMed PMID: 30615663; PubMed Central PMCID: PMCPMC6322729.
45. Van Minh H, Do YK, Bautista MA, Tuan Anh T. Describing the primary care system capacity for the prevention and management of non-communicable diseases in rural Vietnam. The International journal of health planning and management. 2014;29(2):e159-73. Epub 2013/04/05. doi: 10.1002/hpm.2179. PubMed PMID: 23553675.
46. Armstrong-Hough M, Kishore SP, Byakika S, Mutungi G, Nunez-Smith M, Schwartz JI. Disparities in availability of essential medicines to treat non-communicable diseases in Uganda: A Poisson analysis using the Service Availability and Readiness Assessment. PloS one. 2018;13(2):e0192332. Epub 2018/02/09. doi: 10.1371/journal.pone.0192332. PubMed PMID: 29420640; PubMed Central PMCID: PMCPMC5805288.
47. Mutale W, Bosomprah S, Shankalala P, Mweemba O, Chilengi R, Kapambwe S, et al. Assessing capacity and readiness to manage NCDs in primary care setting: Gaps and opportunities based on adapted WHO PEN tool in Zambia. PloS one. 2018;13(8):e0200994. Epub 2018/08/24. doi: 10.1371/journal.pone.0200994. PubMed PMID: 30138318; PubMed Central PMCID: PMCPMC6107121.

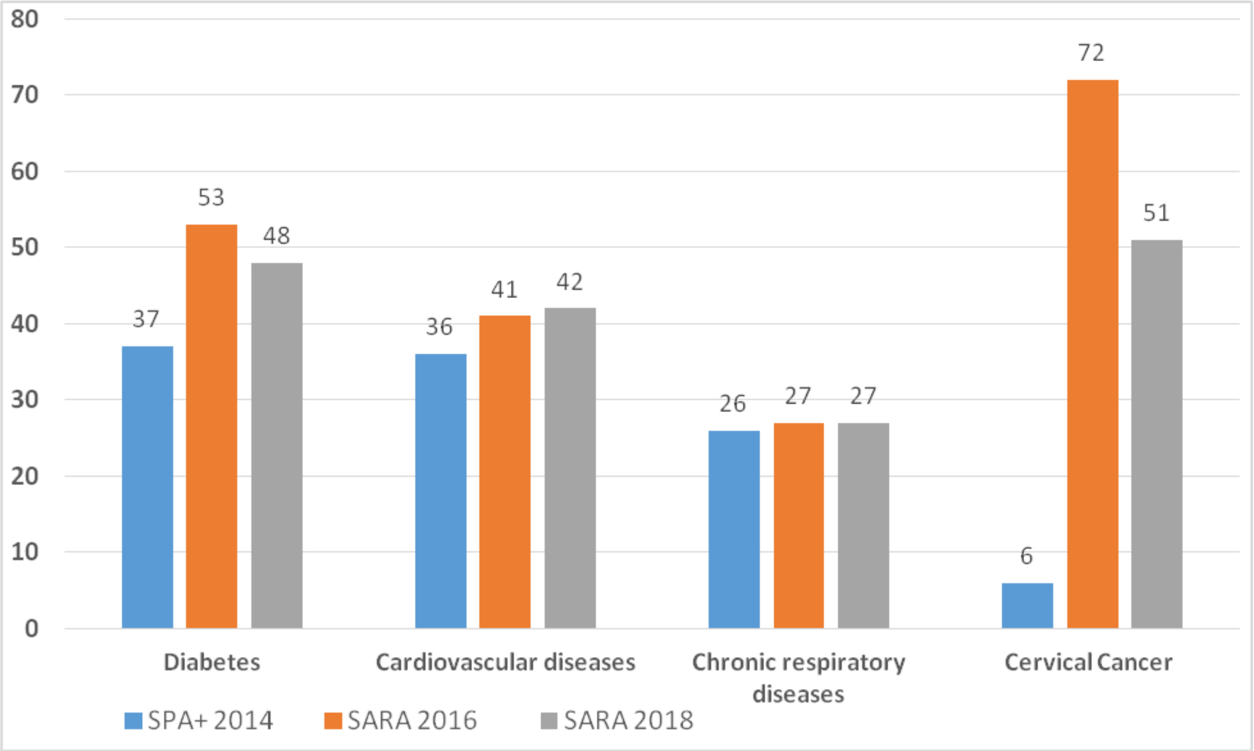

Figure 1: Mean availability of tracer items among facilities that offer diabetes, cardiovascular diseases and chronic respiratory disease services; Ethiopia, SPA+ 2014, SARA 2016 and SARA 2018

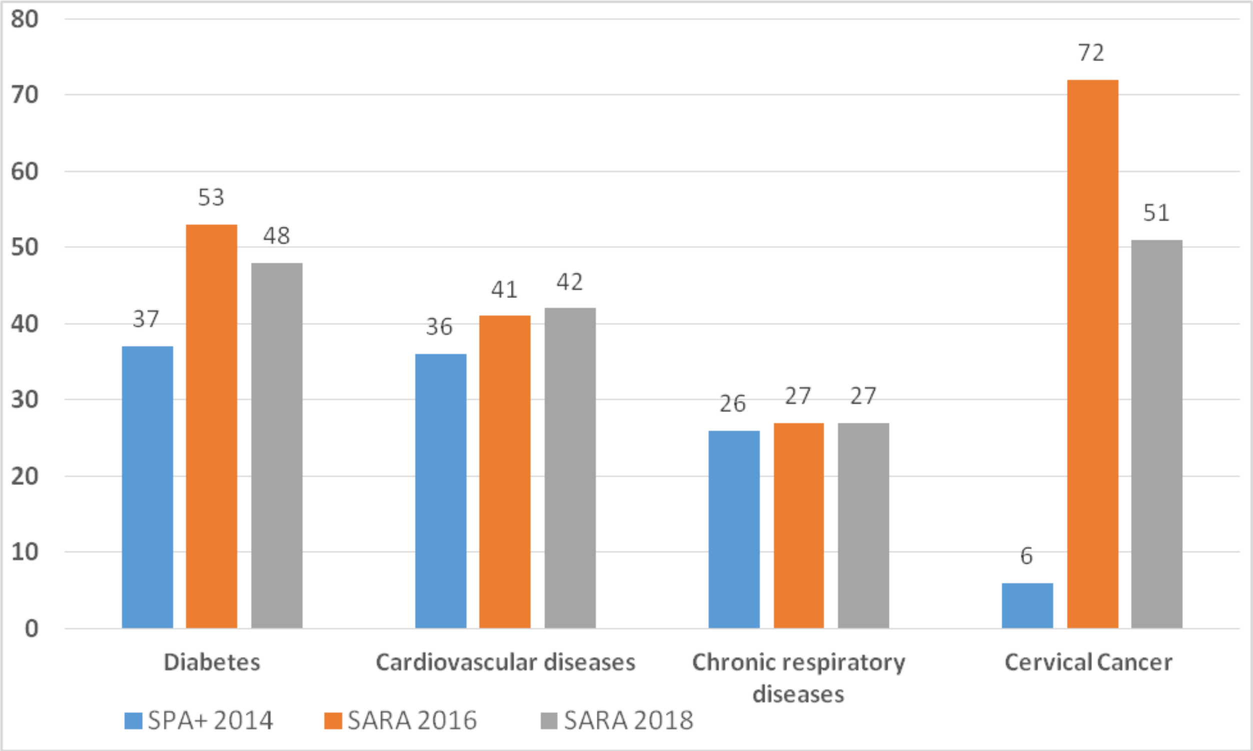

Figure 1: Mean availability of tracer items among facilities that offer diabetes, cardiovascular diseases and chronic respiratory disease services; Ethiopia, SPA+ 2014, SARA 2016 and SARA 2018

Click here to access/download
Supporting Information
22 Nov Table.doc

Health System's Availability and Readiness of Health Facilities for Chronic Non-Communicable Diseases: Evidence from the Ethiopian National Surveys

Atkure Defar[1,2], -Girum Taye Zeleke[1], Della Berhanu[1,3], Ephrem Tekle Lemango[4], Abebe Bekele[1], Kassahun Alemu[2], Sibhatu Biadgilign[5*]

Affiliations

1. Ethiopian Public Health Institute, -Addis Ababa, Ethiopia
2. College of Medicine and Health Science, Institute of Public Health, Department of Epidemiology and Biostatistics, University of Gondar, Gondar, Ethiopia
3. London School of Hygiene & Tropical Medicine, London, UK
4. Maternal, Child Health and Nutrition Directorate, Ministry of Health, Sudan Street, Addis Ababa, Ethiopia.
5. Public Health Nutrition Research Consultant, P.O. Box 24414, Addis Ababa, Ethiopia

Email address:

Atkure Defar: atkuredefar@gmail.com

Girum Taye Zeleke: girumt2000@yahoo.com

Della Berhanu: della.berhanu@lshtm.ac.uk

Ephrem Tekle Lemango: ephremt.lemango@gmail.com

Abebe Bekele: abebe1277belay@gmail.com

Kassahun Alemu: kassalemu@gmail.com

Sibhatu Biadgilign: sibhatu2010@gmail.com

***Corresponding author**

Sibhatu Biadgilign, PhD, MPH, MSc

P.O.Box 24414

Addis Ababa, Ethiopia

Abstract

Introduction: Non-communicable diseases (NCDs) currently cause more deaths than all other causes of deaths. Cardiovascular disease, diabetes, cancer, and chronic respiratory diseases-threaten the health and economies of individuals and populations worldwide. This~~e~~ ~~overall aim of this~~ study aimed~~is~~ to assess health system's availability and readiness of health facilities for chronic non-communicable diseases (NCDs) and describe the changes of~~in~~ ~~the~~ service availability ~~of services~~ for common NCDs in Ethiopia.

Methods: ~~A cross-sectional health facilities-based study was employed and analyzed~~ We used~~ing~~ data from the 2014 Ethiopia Service Provision Assessment Plus (ESPA +) and 2016 and 2018 Service Availability and Readiness Assessment (SARA) surveys which were cross-sectional health facilities-based studies. A total of 873 health facilities in 2014, 547 in 2016, 632 in 2018 were included in the analysis. (ESPA+) and SARA surveys are conducted as a census or a nationally/sub-nationally representative sample of health facilities. Proposition of facilities that offered the service for diabetes, cardiovascular disease, chronic respiratory disease, cancer diseases, mental illness, and chronic renal diseases was calculated to measure health service availability. The health facility service readiness was measured using the mean availably of tracer items that are required to offer the service. Thus, 13 tracer items for diabetes disease, 12 for cardiovascular disease, 11 for chronic respiratory disease and 11 cervical cancer services were used.

Results: The services available for diagnosis and management of diabetes had not shown improvement between 2014 and 2018 with 59% in 2014, 22% in 2016, and 36% in 2018. Similarly for cardiovascular disease, 73%, 41%, 49% in 2014, 2016, and 2018, respectively. Regarding, chronic respiratory disease; 76% in 2014, 45% in 2016 and 53 % in 2018. At the national level in 2014, the mean availability of tracer items for diabetes, cardiovascular disease, chronic respiratory disease, cancer diseases, mental illness and chronic renal diseases were 37%, 36%, 26%, 6%, 7% and 7%, respectively. In 2016, the mean availability of tracer items for diabetes, cardiovascular disease, chronic respiratory disease and cervical cancer were 53%, 41%, 27% and 72%, respectively. Similarly, in 2018, the mean availability of tracer items for diabetes, cardiovascular disease, chronic respiratory disease and cervical cancer were 48%,

42%, 27% and 51%, respectively.

Conclusions: The majority of the health facilities have low and gradual decrement in the availability to provide NCDs services in Ethiopia. Ethiopia. There is a need to increase NCD service availability and readiness at primary hospitals and health centers, and private and rural health facilities where the majority of the population need the receive services.

Formatted: Highlight

Introduction

According to the World Health Organization (WHO) each year the deaths of 40 million people were reported from non-communicable diseases (NCDs), which is equivalent to 70% of all deaths globally [1]. In Ethiopia, NCDs cause 42% of deaths, of which 27% are premature deaths before 70 years of age. The Disability Adjusted Life Years (DALYs) increased from below 20% in 1990 to 69% in 2015 and the incidence and prevalence of NCDs are become rising [2] [3-8]. Furthermore, while the annual number of deaths due to infectious diseases is projected to decline, the total annual number of NCD deaths is projected to increase to 52 million by 2030 [9-11]. The main types of NCDs are cardiovascular diseases (such as heart attack and stroke), cancers, chronic respiratory diseases (such as chronic obstructive pulmonary disease and asthma) and diabetes [1]. Cardiovascular diseases (CVDs) account for most of the annual NCD deaths (17.7 million), followed by cancers (8.8 million), respiratory diseases (3.9 million), and diabetes (1.6 million)[1]. A study of the prevalence of raised blood glucose and diabetes in Ethiopia was 6% and raised blood pressure (SBP > 140 and/or DBP > 90 mmHg) was 15.6%, [12].The death rates of NCDs causes declined by 40% from 1990 to 2015 and also from the 680.9 per 100,000 crude all-cause mortality rate, 286.9 (95% UI: 188.1–423.0) per 100,000 people were due to NCDs[13].

NCDs threaten the progress towards the 2030 Agenda for Sustainable Development, which includes a target of reducing NCD related premature deaths by one-third. In order to attain this target, each country should prioritize and implement cost-effective policies and interventions and provide health services which tackle the rapid surges of this silent epidemic. In low and middle income countries (LMICs), several interacting factors have been associated with high burden of NCDs, including few preventative and early detection services, low availability of essential treatments, and suboptimal organization in the delivery of care[14]. The assessment of the capacity of countries' to prevent and control NCDs conducted by WHO in 2015 also showed major gaps in health system capacity in many LMICs [15]. The absence of evidence-based policies and program with inadequate response at primary care level, thus perpetuating a problem that will become worse with time unless the situation is actively addressed at a critical time [16] especially with- limited data from LMICs on NCD service readiness, availability and utilization in

primary care facilities[17].

In Ethiopia, there are few studies conducted on service availability and readiness for NCDs. These studies, however, are limited as they focus on a particular or selected set of NCDs [18-20]. Other studies in the country also showed that from a total 21 public health facilities surveyed, only 38.1% had all the categories of health professionals as compared to the national standards and 85.2%, of the facilities fulfilled the criteria for basic equipment and 47.7% of the facilities did not fulfill the criteria for infection prevention supplies[21] and this is linked with the availability of essential equipment and drugs, health workers' communication, health care provided, and attitude of health workers had positive association with client satisfaction with various health programs in the health system [21-26].

In Ethiopia, health facilities lack essential logistics and major service provision bottlenecks including non-functionality of the existing district coordination body and technical committees, training gaps, staff shortage, high staff turnover resulting in work related burden, fatigue and poor motivation among service providers [27]. The overall aim of the current study was to assess the availability and readiness of services for common NCDs using Ethiopian Service Provision Assessment (ESPA+) and Service Availability and Readiness Assessment (SARA) surveys in health facilities of Ethiopia.

Methods

Data Source and Study Setting

This study used and analyzed data from the 2014 Ethiopia Service Provision Assessment Plus (ESPA +), and 2016 and 2018 Service Availability and Readiness Assessment (SARA) survey ~~datasets~~. (ESPA+) and SARA surveys are conducted as census or a nationally/sub-nationally representative sample of health facilities that can guide the health sector policy and planning. The surveys were conducted using similar survey methodologies for adapting the questionnaires, training, and pre-testing and data collection. ESPA+ and SARA questionnaires contain information on general and specific service availability and readiness (e.g., types of services offered, basic amenities, equipment, medicines and commodities, diagnostics, trained staff and practice guidelines). The data were collected from all nine regions of Ethiopia namely:

Formatted: Font color: Red

Tigray, Afar, Amhara, Oromia, Somali, Benishangul-gumuz, Southern People Nations and Nationalities (SNNP), Gambella, and Harari. The survey also included the two city administrations, Addis Ababa and Dire Dawa. The Ethiopian health system has primary, secondary and tertiary levels of care. Five health posts, their referral health center and primary hospital comprise the primary health care unit.[28].

Study design

A cross-sectional health facilities-based study was employed in the surveys. The surveys used the SARA methodology that was developed through a joint WHO and the United States Agency for International Development (USAID) collaboration. The method considers, among other thing, the service availability mapping (SAM) tool developed by WHO, and the ESPA+ tool developed by ICF International under the USAID-funded MEASURE DHS (monitoring and evaluation to assess and use results, demographic and health surveys) project[29].

Formatted: Font color: Red

Formatted: Font color: Red

Sampling of health facilities

For ESPA+ 2014, the survey used a stratified random sampling designed to provide representative samples of: health facility types, management authorities, regions, and city administrations. A list of health facilities obtained from each regional health office was used as a sampling frame. This sampling method yielded a total of 1,327 health facilities to be surveyed, which comprised of 223 hospitals, 298 health centers and 321 health posts and 485 private clinics. The sampling method for SARA 2016 and 2018 was also a nationally representative sample stratified by health facility type and managing authority. All health facilities that were in 2016 SARA were selected for the SARA 2018 survey. In the 2016 SARA survey, 705 health facilities were included, with all hospitals (228), 165 health centers, 173 clinics, and 139 health posts. Similarly, in the 2018 SARA survey, a total of 764 health facilities were assessed, which included 303 hospitals, 164 health centers, 165 clinics and 132 health posts. The 2018 SARA survey also included new hospitals that were established after the 2016 SARA survey[29-31].

Formatted: Font color: Red

Formatted: Font color: Red

Formatted: Font color: Red

Formatted: Font color: Red

Data collection tool and procedure

An adapted version of the MEASURE DHS project ~~E~~SPA~~+~~ questionnaire with some additional country specific questions and World Bank service delivery indicator (SDI) were used to collect data on facility characteristics, staff contingents, service availability, referral systems, drugs and equipment, laboratory services, reporting systems, and monitoring and supervision (Refe: Service Availability and Readiness Assessment: An annual Monitoring System for service Delivering). To obtain this information, we interviewed heads of facilities or their delegates or heads of specific sections that were believed to be knowledgeable about the service provision. Inventory and inspection of the guidelines, reports, equipment, drugs and laboratory supplies were also done.

The survey data were collected using Computer-Assisted Personal Interviewing (CAPI) programed using the Census and Survey Processing System (CSPro) 6.3 software. Data were transferred daily to the Ethiopian Public Health Institute server. Feedback was provided to the field team before they ~~left~~ moved to the next selected health facility. The Ethiopian Public Health Institute (EPHI) organized the fieldwork, monitored the data transfer system and carried out extensive supervision over the duration of the fieldwork. Regional coordinators engaged in supervision and actively solved problems faced by the field team.

Data management

The survey data were collected focusing on service readiness and availability for all NCDs in the ESPA+, including diabetes disease, cardiovascular disease, chronic respiratory disease and cancer. To calculate the readiness score, 13 tracer items for diabetes disease, 12 for cardiovascular disease, 11 for chronic respiratory disease and 11 cervical cancer services were used. The availability of the services and readiness of health facilities for diabetes, cardiovascular, and chronic respiratory diseases were investigated in the ESPA+ 2014, and the two recent (2016 and 2018) SARA surveys [29-31]. The service was considered to be available if a facility manages, diagnoses, treats or refers a patient coming to the facility for common NCDs. The facility readiness was estimated based on the availability of tracer items that are necessary to provide the service [32].

Formatted: Highlight

Formatted: Font color: Red

Formatted: Font color: Red

Measurement of variables

The outcome variables in this study were the “availability score” and “readiness score” of the health facilities for common NCDs. We defined facility ‘'availability" as the percentage of facilities in the sample that said they offered the service in question, which considered whether the diagnosis and/or management services were available. Facility "readiness" was a composite measure and was restricted to the subset of facilities that offered the service. The component "domains" that make up the readiness score included trained staff, guideline, ~~and training;~~ equipment/supplies; medicines and commodities ~~and supplies~~, and diagnostics capacity. For each domain, we calculated an index as the mean score of items expressed as a percentage. A readiness score of 50 signifies that, on average, half of the facilities that offered the service had each of the requisite inputs for delivering that service. The calculated indices were compared to an agreed cutoff point of 50%. A facility index that was below 50% was considered as `not ready' to manage NCDs.

Formatted: Font color: Red

Formatted: Font color: Red

Formatted: Font color: Red

Formatted: Font color: Red

Formatted: Font color: Red

Formatted: Font color: Red

Formatted: Font color: Red

Formatted: Font color: Red

Statistical analysis

Data were cleaned and analyzed using Stata 15 (StataCorp, College Station, TX). A descriptive analysis was carried out to summarize results using percentages and proportions, which were then presented in the form of tables and graphs. The analysis for availability and readiness has been done considering the WHO service availably and readiness estimation method [1]. In this study, Fisher's exact test was used to assess the difference in the mean availability of tracer items for various NCDs and across survey years. The analysis did not include health posts since, they do not have standards for NCDs, nor are they supposed to provide curative services for NCDs. The ‘Strengthening the Reporting of Observational Studies in Epidemiology (STROBE) Statement’ to write the manuscript (online supplementary file 1) was followed.

Quality assurance

Quality assurance involved multiple steps along a continuum of training, data collection in the field, and data processing at the central level. Quality assurance began with the recruitment of data collectors and team leaders with a health background. Team leaders played a critical role

in the correct completion of the data collection tools as they reviewed all the questionnaires. The regional coordinators visited and communicated with teams regularly to provide support and help when difficulties arose at individual facilities. The central coordinators at EPHI and members of the Technical Working Group supervised the teams in the field to ensure consistency and quality. When needed, the central data managers telephoned facilities for clarification and to ensure quality.

Ethical consideration

The current study was conducted based on a secondary analysis of existing data sets from the three-waves of data collection (2014, 2016, and 2018). Ethical clearance was obtained from the Scientific Ethical Review Committee (SERC) of the EPHI with reference number of EPHI 6.13/966. Informed verbal consent was obtained from all health facilities' in-charge and ~~sometimes~~ health workers responsible for client services who were present at the facility.

Patient and public involvement

Patients or the public were not involved in the design, or conduct, or reporting, or dissemination plans of this research.

Result

Characteristics of health facilities

In this comprehensive study, a total of 873 health facilities in 2014, 547 in 2016, and 632 in 2018 were included in the analysis. The results are stratified by facility type, managing authority, region, and residence (**Table 1**).

Availability of important components for the management of NCDs

The services available for the diagnosis and/or management of diabetes had not shown improvement between the periods of 2014 to 2018. The availability for diagnosis and/or management services for diabetes was 59%, 22%, and 36% in 2014, 2016, and 2018, respectively. Similarly, the availability for diagnosis and/or management services for cardiovascular disease was 73%, 41%, 49% in 2014, 2016, and 2018, respectively and 76% in

2014, 45% in 2016 and 53% in 2018 for chronic respiratory disease. ~~Only cancer diseases, mental illness and chronic renal diseases service were diagnosed and managed as 23%, 32% and 59% for the survey year of 2014 (**Table 1**). There were regional differences across the availability of health facilities offering the services for the management of NCDs.~~ Mostly, the availability of essential guidelines, trained human power and materials and equipment for the management of NCDs services varied across the survey years~~ and regions~~. Detail description is illustrated in **Table 1.**

Health facility readiness for the management of NCDs

Service readiness was measured generally by the availability of selected tracer items. In all categories of diseases, the readiness was checked by the availability of guideline and at least 1 trained staff as necessary tracer items to provide the service. Additionally, the analysis considered the availability items listed in **Table 2**. The general service readiness for cancer disease, mental illness, and chronic renal disease were measured based on the availability of guideline and at least 1 trained staff as a common tracer items.

Service readiness is estimated among those who offered the services and was made by assessing the four domains: staff and guidelines, equipment, diagnostics, and medicines and Commodities. The bar graph shows the trend on overall readiness using the availability of tracer items for three of the selected NCDs (diabetes, cardiovascular, cervical cancer and chronic respiratory disease), which showed an improvement across different periods. We used 12 tracer items that are necessary to provide diabetes service in 2014, whereas 13 tracer items were used for both 2016 and 2018 surveys. On average, for each survey years, facilities had 4, 6, and 6 tracer items with overall readiness score of 37%, 53%, and 48%, respectively. This showed improvement over the period between 2014 and 2016. The overall readiness for cardiovascular diseases (of the total 12) did not show improvement over time, 36%, 41%, and 42% during, 2014 (10 tracer items), 2016 (of 12 tracer items), and 2018 (12 tracer items), respectively (**Figures 1**).

Of the 11 tracer items that are necessary to provide diabetes service, on average facilities had 3 tracer items with an overall readiness score of 26%. There were no changes over time 2016

Formatted: Font color: Red

Formatted: Font color: Red

Formatted: Font color: Red

(27%) and 2018 (27%), with the mean tracer item availability of 3 out of 11 items. At the national level, the mean availability of tracer items for diabetes, cardiovascular disease, chronic respiratory disease, cancer diseases, mental illness, and chronic renal diseases were 37%, 36%, 26%, 6%, 7% and 7%, respectively in 2014. In 2016, the mean availability of tracer items for diabetes, cardiovascular disease, chronic respiratory disease and cervical cancer were 53%, 41%, 27% and 72%, respectively. Similarly, the mean availability of tracer items for diabetes, cardiovascular disease, chronic respiratory disease, and cervical cancer were 48%, 42%, 27% and 51%, respectively in 2018 **(Table 2).**

Formatted: Font color: Red

Formatted: Font color: Red

Changes in preparedness for common non-communicable diseases

Table 3 shows the differences in the mean availability of tracer item in percent for NCDs service availability between assessment years at the national level. In this study, there was an improvement from 2014 to 2016 in terms of the availability of tracer items with 16% for diabetes (statistically significant), 5% cardiovascular disease, and 1% chronic respiratory disease. However, from 2016 to 2018 survey year, there is a decrease for diabetes with 5% and 21% for cervical cancer (statistically significant) and showed almost no change for cardiovascular and chronic respiratory disease (**See table 3).** The change in mental health and chronic renal disease service readiness were not assessed since we have only one time data for the 2014.

Discussion

The study highlighted and revealed that major aspects of addressing service availability and readiness ~~and readiness~~ of health facilities for common NCDs services are compromised in ~~the country~~ Ethiopia across various periods. ~~The study provides a comprehensive and in-depth analysis of availability and readiness of health facilities to provide services for NCDs in Ethiopia.~~ The availability for diagnosis and/or management services for diabetes was 59%, 22%, and 36% in 2014, 2016, and 2018, respectively and also for cardiovascular disease, 73%, 41%, and 49 % in 2014, 2016, and 2018, for chronic respiratory disease; 76% in 2014, 45% in 2016 and 53 % in 2018 respectively. Similar finding has been documented in South Africa, which identified that the need to improve availability of NCD-trained nurses, functional equipment, and medication

Formatted: Font color: Red

[33]. In Ugandan study, none of the facilities included met the WHO package of essential non-communicable (PEN) standards for essential tools and medicines to implement effective NCD interventions [34].

Studies in Ethiopia documented that the overall prevalence of chronic NCDs was 8.9% and specific prevalence were 0.5% for diabetes mellitus (DM), 2.6% for hypertension, 3.0% for cardiovascular diseases, 1.5% for asthma and 2.7% for mental illness in Gilgel Gibe Field Research Center[35]- and national cardiovascular disease prevalence of 15%, cancer and chronic obstructive pulmonary disease prevalence of 4% each, and diabetes mellitus prevalence of 2%[36].

Health facilities included in this study were not well equipped to provide services for, diabetes, cardiovascular disease, and chronic respiratory disease. The trend was similar across the survey years. Diabetes had the lowest service availability, but the highest readiness score to provide service among the common NCDs. The availability of guidelines and at least one trained staff is minimal to deliver the management of diabetes, cardiovascular diseases, cervical cancer, and chronic respiratory disease services. According to the WHO report on the health care capacity for NCD management, 2020, evidence-based guidelines for the management of NCDs need to be developed and broadly implemented to ensure appropriate diagnosis, referral and treatment. Also reported that, globally, only 48% of countries reported having evidence-based guidelines, protocols or standards for the management of all four NCDs through a primary care approach. In contrary to our study, the report revealed that, guidelines for diabetes were most widely available (84%), followed by CVDs (77%), cancer (70%) and CRDs (64%). Correspondingly, countries were most likely to have guidelines for diabetes that included referral criteria and were utilized in at least 50% of health care facilities, with 80% of countries in upper-middle-income countries reporting having such guidelines (Refe: WHO, Health Care capacity for NCD management, 20202). The decline in the trends of service availability and readiness could be the fact that the skilled staff turnover is high form the rural to urban areas. Also, the primary health care system might not be ready in terms of infrastructure, staffing and medicine and commodities which is supposed to provide the service which makes the denominator up in calculating readiness.

Formatted: Font color: Red

Formatted: Font color: Red

Formatted: Font color: Red

Formatted: Font color: Red

Formatted: Font: Times New Roman, 12 pt, Font color: Custom Color(RGB(34,34,34)), English (United Kingdom), Pattern: Clear (White)

Formatted: Font: Times New Roman, 12 pt, Font color: Custom Color(RGB(34,34,34)), Pattern: Clear (White)

Formatted: Font: Times New Roman, 12 pt, Font color: Custom Color(RGB(34,34,34)), Pattern: Clear (White), Highlight

Formatted: Font: Times New Roman, 12 pt, Font color: Custom Color(RGB(34,34,34)), Pattern: Clear (White), Highlight

Formatted: Font: Times New Roman, 12 pt, Font color: Custom Color(RGB(34,34,34)), Pattern: Clear (White)

Formatted: Font: Times New Roman, 12 pt, Font color: Custom Color(RGB(34,34,34)), Pattern: Clear (White)

Formatted: Font: Times New Roman, 12 pt, Font color: Custom Color(RGB(34,34,34)), Pattern: Clear (White)

Formatted: Font: Times New Roman, 12 pt, Font color: Custom Color(RGB(34,34,34)), Pattern: Clear (White)

Formatted: Font: Times New Roman, 12 pt, Font color: Custom Color(RGB(34,34,34)), Pattern: Clear (White)

Formatted: Font: Times New Roman, 12 pt, Font color: Custom Color(RGB(34,34,34)), Pattern: Clear (White)

Formatted: Font: Times New Roman, 12 pt, Font color: Custom Color(RGB(34,34,34)), Pattern: Clear (White)

Formatted: Font: Times New Roman, 12 pt, Font color: Custom Color(RGB(34,34,34)), Pattern: Clear (White)

SimilarlyOur study is consistent with the, in a study conducted in Uganda, less than a half (46%) of the health facilities had hypertension guidelines with significant differences observed across health facility levels [37] which is one of the core aspects of the management of the chronic diseases. Similarly, a study done in Tanzania showed that in many health facilities, guidelines, diagnostic equipment, and first-line drug therapy for the primary care of NCDs were inadequate, and management, training, and reporting systems were weak [16]. Likewise, in the recent Tanzanian national survey, out of 725 health facilities involved in the study, only 28% of the assessed facilities were considered prepared for the outpatient primary care of hypertension. About 9% and 42% of the assessed facilities reported to have at least one trained staff and guidelines for hypertension, respectively [38]. In a Ghanaian study, the findings highlight the unavailability of essential medicines required for adequate management of NCDs reflected in the limited capacity at the various levels of care [39]. More importantly, there are challenges for healthcare delivery system: first the double burden of communicable diseases and non-communicable diseases such as diabetes and cardiovascular disease; the burden that chronic conditions impose on health systems and multiple chronic conditions and lastly burden on the primary healthcare [40]. According to the WHO recommendations, training of the health workforce and strengthening the capacity of the health systems, particularly at primary care level and policy options to address the prevention and control of NCDs are all priorities [41].

From our findings, trained human resources were inadequate for addressing and managing NCDs in Ethiopia. Consistent result was documented in Tanzanian health facilities for outpatient primary care study, in which trained health workers for NCDs were inadequate, and management, training, and reporting systems were weak. Only one of the 24 health facilities surveyed reported any training in the past year for hypertension or diabetes [16]. According to the national survey in Tanzania, about 26% and 13% of health facilities had guidelines and trained staff for NCDs, respectively [42]. Coupled with this, in a Ghanaian study, inadequate staffing was a major impediment for the capacity of primary healthcare facilities to serve NCD-related health needs [39]. The capacity of the primary care system in Vietnam was inadequate to serve the NCD-related health needs of the population [43]. Similarly, a study documented in Ethiopia showed that encouraging actions and results on NCDs in terms of political commitment, however, there is a gap as shown by the limited availability of resources for NCDs

Formatted: Font color: Red

Formatted: Normal (Web), Left

Formatted: Font color: Red

Formatted: Font color: Red

Formatted: Font color: Red

Formatted: Font: Lato, 9 pt, Font color: Custom Color(RGB(22,22,20)), English (United Kingdom), Pattern: Clear

Formatted: Font color: Red

Formatted: Font color: Red

Formatted: Font color: Red

Formatted: Font color: Red

prevention and treatment services at the primary health care level. Shortage of essential NCD drugs and diagnostic facilities and lack of treatment guidelines are major challenges[2].

This study showed that the majority of the services for chronic disease management occurred in a referral hospital, general hospital, and primary hospital, unlike the primary health care facilities. Although, people with, or at risk of developing, NCDs require long-term care that is proactive, patient- centered, community-based and sustainable. Such care can be delivered equitably only through robust health systems founded on strong primary health care towards the attainment of universal health coverage (Refe: WHO, Health Care capacity for NCD management, 20202).

This is also a case in Uganda whereby limiting the supply of anti-hypertensive medicines to higher-level health facilities is incongruent with the provision of high quality, chronic care for persons with hypertension in lower health facilities. In the same way, lower-level health facilities, where the population is expected to receive primary health care, should be expected to stock essential medicines for treating non-communicable diseases[44]. A similar finding is also documented in Zambia where nearly all primary health facilities studied did not meet the minimum threshold to manage NCDs [45] according to the WHO recommendations [41].

Our study has some limitations. Firstly, the sampling frames used in the surveys is limited to health ~~facilities~~ centers/health posts included in the country master health facility list and some other health facilities especially those recently constructed might not be included. Secondly, the cross-sectional nature of the study limits its value to explain the relative importance of some of the factors to be considered. In the same time, such kind of surveys conducted in some cases, some of the variables like hospitals, focusing more governmental and urban health facilities seems oversampled. Thirdly, other concern might be regional variations in the availability and management of NCDs across the regions of the country. Lastly, as with the nature of the data, p-values estimation should be regarded as exploratory rather than for testing hypothesis.

Formatted: Line spacing: 1.5 lines, Pattern: Clear

Formatted: Font: Times New Roman, 12 pt, Not Bold, Font color: Auto

Formatted: Font: Lato, 9 pt, Font color: Custom Color(RGB(22,22,20)), English (United Kingdom)

Formatted: Font color: Red

Formatted: Font color: Red

Formatted: Font color: Red

Formatted: Font color: Red

Formatted: Font color: Red

Formatted: Font color: Red

Despite the above limitations, our study has several strengths. Firstly, this study undertook in a representative sample of health facilities as it is collected from health facilities in the country, so it might be generalisable to all facilities in the country. Secondly, the survey tools were using standardized WHO-recommended and validated data collection instrument, training and pre-testing of the tools was rigours in nature, the involvement of policymakers and health programme managers, approval makes for endorsement of the finding of the study for improving quality and management of NCDs carried out in the health facilities. In addition to this, it is a nationally representative sample and wealth of data to overlook the temporal pattern across the years were taken in which the result demonstrates the real situation of the health facilities regarding the health system's availability and readiness of the services for NCDs in Ethiopia.

Formatted: Font color: Red

Formatted: Font color: Red

The ~~policy implications of the findings is that the~~ temporal trend identified for the common non-communicable disease service availability observed from 2014 to 2018 should be used~~has important implications for~~to ~~deciding the~~ prioritiz~~ey~~ ~~of the~~ health care programs and scaling up interventions for NCDs in Ethiopia and other developing countries. Health system policy shall~~ould~~ given due attention to NCDs service availability. In addition, from each specific NCDs~~S~~, the government has to focus the attention towards prioritizing ~~the~~ each specific disease in descending order for chronic respiratory disease, cardiovascular disease and diabetes while providing and expanding the NCDs service provision in government/public health facilities as well as focusing on rural area and primary health care service where the majority of the community members served for other services outlet~~s~~. Similarly, it will add a value on the existing NCDs prevention strategies that would be strengthened through updating the priority disease that need further attention and implication.

Conclusions

The majority of the health facilities have low and gradual decrement in the availability to provide NCDs services in Ethiopia. However, there is stability and incremental on readiness score across the type of NCDs and importantly, more attention is needed on facility readiness to provide cervical cancer, mental illness, and chronic renal diseases services. There is a need to increase NCD service availability and readiness at the primary health care system, giving special

attention to the primary hospitals and health centers, and private and rural health facilities where the majority of the population receive services. Basic diagnostic equipment and medication also need to be made available. Management of NCDs can also be improved by providing continuous training for primary health care workers. Opportunities for providing integrated care for NCDs should receive strong consideration, allowing for efficient use of the available resources at primary health care in low-income countries such as Ethiopia.

Declarations

Ethics approval and consent to participate

The current study was conducted based on a secondary analysis of existing data sets from the three-wave of data collection (2014, 2016, and 2018). Ethical clearance was obtained from the scientific Ethical Review Committee (SERC) of the Ethiopian Public Health Institute (EPHI 6.13/966). Informed verbal consent was obtained from all health facilities in-charge and sometimes health workers responsible for client services who were present at the facility. Informed consent was obtained from the participant.

Consent for publication

Not applicable.

Availability of data and materials

The author of this manuscript has no right to share or put the data on a public data repository to be accessible by all who need the data, It can only be accessed through a formal request to the Ethiopian Public Health institute, National Data Management Centre.

~~Data will be available from the corresponding author upon reasonable request~~

Competing Interests

The Authors declare that they don't have any competing interests.

Authors' Contributions

Formatted: Font: Times, 12 pt

Formatted: Space Before: 0 pt, After: 6 pt, Line spacing: 1.5 lines, Don't adjust space between Latin and Asian text, Don't adjust space between Asian text and numbers

Conceptualization, AD, GT , BD, ET, AB, KA and SB; Data curation, Atkure Defar, Girum Taye Zeleke and Kassahun Alemu ; Formal analysis, Atkure Defar, Girum Taye Zeleke, Della Berhanu, Kassahun Alemu and Sibhatu Biadgilign; Investigation, Atkure Defar, Girum Taye Zeleke, Della Berhanu, Ephrem Tekle Lemango, Abebe Bekele and Sibhatu Biadgilign; Methodology, Atkure Defar, Girum Taye Zeleke, Della Berhanu, Ephrem Tekle Lemango, Abebe Bekele, Kassahun Alemu and Sibhatu Biadgilign; Project administration, Atkure Defar; Resources, Atkure Defar and Sibhatu Biadgilign; Supervision, Della Berhanu, Ephrem Tekle Lemango , Abebe Bekele, Kassahun Alemu and Sibhatu Biadgilign; Validation, Girum Taye Zeleke and Sibhatu Biadgilign; Visualization, Ephrem Tekle Lemango, Abebe Bekele, Kassahun Alemu and Sibhatu Biadgilign; Writing – original draft, Atkure Defar, Girum Taye Zeleke, Della Berhanu, Ephrem Tekle Lemango, Abebe Bekele, Kassahun Alemu and Sibhatu Biadgilign; Writing – review & editing, Atkure Defar, Girum Taye Zeleke, Della Berhanu, Ephrem Tekle Lemango , Abebe Bekele, Kassahun Alemu and Sibhatu Biadgilign.

Acknowledgement

We acknowledged to the Ministry of Health and the Ethiopian Public Health Institute for providing the data. As well, we are highly indebted to appreciate the contribution of study participants as well as data collectors. The surveys were undertaken by the Ethiopian Public Health Institute (EPHI), which is the research arm of the Ethiopian Ministry of Health (FMoH). They were also done in collaboration with various organizations. We would like to thank , ~~For 2014 ESPA+,~~ ICF International provided technical assistance, ~~-and the survey was funded by~~ the United States Agency for International Development (USAID), World Bank, Irish Aid, WHO and UNICEF for their support during the 2014 ESPA+~~. For 2016 and 2018 surveys, technical support was provided by the~~, World Health Organization, World Bank and Global Fund for their support during the 2016 and 2018 surveys.~~. World Health Organization provided the financial support for the survey in 2016 and The World Bank provided financial support for the 2018 surveys.~~

Formatted: Font color: Red

Formatted: Font color: Red

Formatted: Font color: Red

Formatted: Font color: Red

Formatted: Font color: Red

Disclaimer

The views, findings and conclusions represented in this article are those of the authors and do not necessarily represent the official views of the institutions with which they are affiliated.

Funding

The funders had no role in study design, data collection and analysis, decision to publish, or preparation of the manuscript.", We also would like to notify that the authors received no specific funding for this work

~~The study did not receive any specific grant from any funding agency in the public, commercial or not-for-profit sectors.~~

Formatted: Font: Not Italic

Formatted: Space Before: 0 pt, After: 6 pt, Line spacing: 1.5 lines, Don't adjust space between Latin and Asian text, Don't adjust space between Asian text and numbers

Formatted: Font: (Default) Calibri, 12 pt, Not Italic

Formatted: Font: (Default) Calibri, 12 pt, Not Italic, English (United States)

Formatted: Font: (Default) Calibri, 12 pt, Not Italic

Reference

1. WHO. Key facts on noncommunicable diseases. World Health Organization(WHO) (Available at https://www.who.int/news-room/fact-sheets/detail/noncommunicable-diseases ; accessed at January 30, 2019) (2018).
2. Shiferaw, F., *et al.* Non-communicable Diseases in Ethiopia: Disease burden, gaps in health care delivery and strategic directions. *Ethiop. J. Health Dev* **32**, 00-000 (2018).
3. Demilew, Y.M. & Firew, B.S. Factors associated with noncommunicable disease among adults in Mecha district, Ethiopia: A case control study. *PloS one* **14**, e0216446 (2019).
4. Abebe, S.M., *et al.* The prevalence of non-communicable diseases in northwest Ethiopia: survey of Dabat Health and Demographic Surveillance System. *BMJ open* **7**, e015496 (2017).
5. Prevett, M. Chronic non-communicable diseases in Ethiopia-a hidden burden. *Ethiopian journal of health sciences* **22**, 1 (2012).
6. Giday, A., Wolde, M. & Yihdego, D. Hypertension, obesity and central obesity in diabetics and non diabetics in Southern Ethiopia. *Ethiopian Journal of Health Development* **24**(2010).
7. Abebe, S.M., Berhane, Y., Worku, A. & Getachew, A. Prevalence and associated factors of hypertension: a crossectional community based study in northwest ethiopia. *PloS one* **10**, e0125210 (2015).
8. Girum, T., Mesfin, D., Bedewi, J. & Shewangizaw, M. The burden of noncommunicable diseases in Ethiopia, 2000–2016: analysis of evidence from global burden of disease study 2016 and global health estimates 2016. *International journal of chronic diseases* **2020**(2020).
9. UNDESA. World Population Prospects: The 2012 Revision, Key Findings and Advance Tables. United Nations, Department of Economic and Social Affairs Population Division(UNDESA) . Working Paper No. ESA/P/WP.227 (2013).
10. Mathers, C.D. & Loncar, D. Projections of global mortality and burden of disease from 2002 to 2030. *PLoS medicine* **3**, e442 (2006).
11. WHO. World Health Organization. Projections of mortality and causes of death, 2015 and 2030 (http://www.who.int/healthinfo/global_burden_disease/projections/en/, http://www.who.int/gho/ncd/mortality_morbidity/en/, accessed 8, January 2019) (2014).
12. EPHI. Summary Report On Ethiopia Steps Survey On Risk Factors For Chronic Noncommunicable Diseases And Prevalence Of Selected NCDs. Ethiopian Public Health Institute (EPHI) and Ministry of Health (MOH) (2016).
13. Misganaw, A., *et al.* National mortality burden due to communicable, non-communicable, and other diseases in Ethiopia, 1990-2015: findings from the Global Burden of Disease Study 2015. *Population health metrics* **15**, 29 (2017).
14. Ali, M.K., *et al.* Systems and capacity to address noncommunicable diseases in low- and middle-income countries. *Science translational medicine* **5**, 181cm184 (2013).
15. WHO. Assessing national capacity for the prevention and control of noncommunicable diseases: report of the 2017 global survey. Geneva: World Health Organization(WHO). Licence: CC BYNC-SA 3.0 IGO. (2018).
16. Peck, R., *et al.* Preparedness of Tanzanian health facilities for outpatient primary care of hypertension and diabetes: a cross-sectional survey. *The Lancet. Global health* **2**, e285-292 (2014).
17. Duong, D.B., Minh, H.V., Ngo, L.H. & Ellner, A.L. Readiness, Availability and Utilization of Rural Vietnamese Health Facilities for Community Based Primary Care of Non-communicable Diseases: A CrossSectional Survey of 3 Provinces in Northern Vietnam. *International journal of health policy and management* **8**, 150-157 (2019).
18. Getachew, T., *et al.* Service availability and readiness for major noncommunicable diseases at health facilities in Ethiopia *Ethiop. J. Health Dev* **31**, 384-390 (2017).
19. Bekele, A., *et al.* Service availability and readiness for diabetes care at health facilities in Ethiopia. *Ethiop. J. Health Dev* **31**, 110-118 (2017).
20. Gelibo, T., *et al.* Availability and readiness of services for cancer care at health facilities in Ethiopia: Implication for action. *Ethiop. J. Health Dev* **31**, 391-396 (2017).

21. Abazinab, S., Woldie, M. & Alaro, T. Readiness of Health Centers and Primary Hospitals for the Implementation of Proposed Health Insurance Schemes in Southwest Ethiopia. *Ethiopian journal of health sciences* **26**, 449-456 (2016).
22. Kumsa, A., Tura, G., Nigusse, A. & Kebede, G. Satisfaction with emergency obstetric and new born care services among clients using public health facilities in Jimma Zone, Oromia Regional State, Ethiopia; a cross sectional study. *BMC pregnancy and childbirth* **16**, 85 (2016).
23. Asrese, K. Quality of intrapartum care at health centers in Jabi Tehinan district, North West Ethiopia: clients' perspective. *BMC health services research* **20**, 439 (2020).
24. Deribew, A.*, et al.* Capacity of health facilities for diagnosis and treatment of HIV/AIDS in Ethiopia. *BMC health services research* **18**, 535 (2018).
25. Deribew, A.*, et al.* Health system capacity for tuberculosis care in Ethiopia: evidence from national representative survey. *International journal for quality in health care : journal of the International Society for Quality in Health Care* **32**, 306-312 (2020).
26. Fisseha, G., Berhane, Y., Worku, A. & Terefe, W. Quality of the delivery services in health facilities in Northern Ethiopia. *BMC health services research* **17**, 187 (2017).
27. Fenta, E.H.*, et al.* Landscape analysis of nutrition services at Primary Health Care Units (PHCUs) in four districts of Ethiopia. *PloS one* **15**, e0243240 (2020).
28. FMoH. Health Sector Transformation Plan: 2015/16-2019/20. Addis Ababa: Ethiopia. Federal Democratic Republic of Ethiopia Ministry of Health (FMoH) (2015).
29. EPHI. Ethiopia Service Provision Assessment Plus (ESPA+) Survey 2014. Ethiopian Public Health Institute (EPHI) and Ethiopian Ministry of Health (MOH) (2014).
30. EPHI. Ethiopia Service Availability and Readiness Assessment (SARA) 2016 Summary Report. Ethiopian Public Health Institute (EPHI) and Ministry of Health (MOH) (2017).
31. EPHI. Ethiopia Service Availability and Readiness Assessment (SARA) 2018 Final Report. Ethiopian Public Health Institute (EPHI) and Ministry of Health (MOH) (2018).
32. WHO. Service Availability and Readiness Assessment (SARA). An annual monitoring system for service delivery Reference Manual. Version 2.1 September 2013. World Health Organization (WHO) WHO/HIS/HSI/RME/2013/1 (2013).
33. Maimela, E.*, et al.* Interventions for improving management of chronic non-communicable diseases in Dikgale, a rural area in Limpopo Province, South Africa. *BMC health services research* **18**, 331 (2018).
34. Rogers, H.E., Akiteng, A.R., Mutungi, G., Ettinger, A.S. & Schwartz, J.I. Capacity of Ugandan public sector health facilities to prevent and control non-communicable diseases: an assessment based upon WHO-PEN standards. *BMC health services research* **18**, 606 (2018).
35. Muluneh, A.T.*, et al.* Population based survey of chronic non-communicable diseases at gilgel gibe field research center, southwest ethiopia. *Ethiopian journal of health sciences* **22**, 7-18 (2012).
36. WHO. Noncommunicable diseases country profiles 2011. Geneva: World Health Organization(WHO); 2011. p. 207 p. (2011).
37. Musinguzi, G.*, et al.* Capacity of Health Facilities to Manage Hypertension in Mukono and Buikwe Districts in Uganda: Challenges and Recommendations. *PloS one* **10**, e0142312 (2015).
38. Bintabara, D. & Mpondo, B.C.T. Preparedness of lower-level health facilities and the associated factors for the outpatient primary care of hypertension: Evidence from Tanzanian national survey. *PloS one* **13**, e0192942 (2018).
39. Nyarko, K.M.*, et al.* Capacity assessment of selected health care facilities for the pilot implementation of Package for Essential Non-communicable Diseases (PEN) intervention in Ghana. *The Pan African medical journal* **25**, 16 (2016).
40. Bischoff, A., Ekoe, T., Perone, N., Slama, S. & Loutan, L. Chronic disease management in Sub-Saharan Africa: whose business is it? *International journal of environmental research and public health* **6**, 2258-2270 (2009).
41. WHO. Global action plan for the prevention and control of noncommunicable diseases 2013-2020. World Health Organization (WHO). ISBN 978 92 4 150623 6 (2013).
42. Shayo, F.K. & Bintabara, D. Are Tanzanian health facilities ready to provide management of chronic respiratory diseases? An analysis of national survey for policy implications. *PloS one* **14**, e0210350 (2019).

43. Van Minh, H., Do, Y.K., Bautista, M.A. & Tuan Anh, T. Describing the primary care system capacity for the prevention and management of non-communicable diseases in rural Vietnam. *The International journal of health planning and management* **29**, e159-173 (2014).
44. Armstrong-Hough, M.*, et al.* Disparities in availability of essential medicines to treat non-communicable diseases in Uganda: A Poisson analysis using the Service Availability and Readiness Assessment. *PloS one* **13**, e0192332 (2018).
45. Mutale, W.*, et al.* Assessing capacity and readiness to manage NCDs in primary care setting: Gaps and opportunities based on adapted WHO PEN tool in Zambia. *PloS one* **13**, e0200994 (2018).

Dear Charu C Garg (Ph.D)
Academic Editor
PLOS ONE Journal

Thanks for the comments, concerns, and revision points you and the three reviewers have provided to this manuscript titled "**Health System's Availability and Readiness of Health Facilities for Chronic Non-Communicable Diseases: Evidence from the Ethiopian National Surveys**" number PONE-D-22-03523. We have now addressed most of the comments and amended, you could find in the file "revised manuscript track changes" and highlighted in red. And we have revised the manuscript according to the PLOS ONE style and guideline and captions are corrected.

Regarding the funding, we would like to disclose that no fund was obtained for this study and would like to write in the manuscript in the following way. "*The funders had no role in study design, data collection and analysis, decision to publish, or preparation* of the manuscript." The authors received no specific funding for this work."

The data availability could be described like this: - *Authors of this manuscript has not right to share or put the data on a data repository to be accessible by all who need the data. The data can be accessed through a formal request to the Ethiopian Public Health institute and it's also available at the Website of ICF international.*

The acknowledgment section is corrected accordingly. "*We acknowledged to the Ministry of Health and the Ethiopian Public Health Institute for providing the data. As well, we are highly indebted to appreciate the contribution of study participants as well as data collectors. The surveys were undertaken by the Ethiopian Public Health Institute (EPHI), which is the research arm of the Ethiopian Ministry of Health (FMoH). They were also done in collaboration with various organizations. We would like to thank, for ICF International provided, the United States Agency for International Development (USAID), World Bank, Irish Aid, WHO and UNICEF for their technical assistance during the 2014 ESPA+. Ands the World Health Organization, World Bank and Global Fund during the 2016 and 2018 surveys*."

Additional Editor Comments (if provided):

Thanks for submitting this paper on an important area to this journal. The paper is interesting, but can benefit from some revisions as suggested by the 3 reviewers and also my own comments in the attached document.

The methods section needs to have more clarity on defining the tracer elements. Put it in the context of WB SDI and explain the reasons for variables that were chosen as tracer values. In the tables the n values must be provided when the % are being presented for each variable.

RESPONSE:
Tracer indicators are items that can measure the specific domain of assessing service specific availability and readiness in objective information about whether or not a facility meets the required conditions to support provision of basic or specific services with a consistent level of quality and quantity. For instance, tracer items for availability and readiness of the service include trained staff, guidelines, equipment, diagnostic capacity, and medicines and commodities.

Reviewer #1:
The paper covers changes in health facility and readiness in managing NCDs in Ethiopia using national SARA surveys and interestingly reports a decline in many parameters. While potentially relevant for readers, it needs to address some critical issues better.
Abstract:

The results especially the for availability of tracer items need to be given for each disease by year for easy understanding.

RESPONSE
Thanks, you for the comment. We have now revised the abstract section considering the availability of tracer items. Some disease such as mental health and chronic renal disease have not been assessed during the 2016 and 2018. Thus, we are not able to compare with the 2014 survey result. General cancer service readiness is also not assessed during these periods rather it was specific for cervical cancer service readiness. We have described this under the limitation section.

Main paper:
Introduction: 40 million people were not reported to have died from NCDs but were estimated to have died. Premature deaths due to NcDs are those between 30-69 years and not below 70 years.
As the paper focuses on comparison between the three surveys, it is critical that reader knows the three survey methodology better. It will be good to tabulate details of the three surveys (sample size, sampling, tools used, other relevant measurement issues etc,).

RESPONSE
Thank you for the concern. There is NOT much methodological difference among the three surveys, but we have added more methodological details about the three surveys. This is also indicated in the Method section under Data source and Study setting and study design subsections.
The 2018 SARA (768) was conducted in the same health facilities that were included in 2016 (705). The difference is due to the facilities that the surveys were considering all hospitals to be assessed. Thus, the newly constructed and upgraded health facilities make a difference in the number. We have indicated this under the last two line of the paragraph in the Sampling and health facility sub-section.

The number and proportion of health facilities changed in the surveys in terms of primary/secondary/tertiary. Could these have affected the results and the decline is a reflection of choice of facilities than the decline. The authors have to ensure that measurement issues are not causing a bias in terms of decline of service availability and readiness. The results should also be presented by the level of facilities. Have they used a weighted prevalence to adjust for these differences?

RESPONSE
Thank you for the concern. This is not a repeated cross sectional study. The study is conducted in a representative sample of all hospitals and selected health centres and health posts. Thus, we wouldn't expect the same health facilities to be included in different survey time. The analysis also considered the proportion which would NOT be affected by sampling since all survey considered random sampling from the list of Ethiopian health facilities as a sampling frame.

Readiness was only measured in subset of facilities that offered services. I think the denominator has to be consistent as all facilities.

RESPONSE
The denominator for service availability is all facility that is assessed in the survey, but for the readiness first that facility should provide/offer the service. That is according to the WHO Service Availably and Readiness Assessment guide.
It is not clear how the readiness score can be that half of the facilities that offered the service had each of the input. OR was it that at least 50% of the inputs were available in a facility which had a readiness score of 50?

RESPONSE
We have not added more explanation to make it clearer. A readiness score of 50% is on average that 50% of the health facilities assessed have all the necessary trace items to provide the service. This is just to make clearer about how the means score is calculated and interpreted.

Table 1 - Region can be deleted. If needed a map may be provided with location of studies facilities to ensure that these are geographically well spread. As mental health and CKD were only seemed to have been assessed in 2014, these can be deleted.

RESPONSE
Thank you for the comment. We accepted the comments and have now removed the region and mental health and chronic kidney disease (Tables 1).

Discussion has to really focus on whether the decline is real or because of selection and measurement bias. If real, then a better explanation of the why this occurred based on the

authors knowledge of context. There is no point comparing these findings with other countries as they are not very relevant.

RESPONSE

Thank you so much for the comment. We want to assure that there wasn't any measurement bias that makes the decline in service availability and readiness, since we have used the standard survey data that have been collected in different times. We believe that all the data collection tool, measurement approach is up to the standard. In addition, we have added few contextual explanations about why the decline in service readiness.

Reviewer #2:

This is an important analysis in the given context and the use of standardised survey data makes it comparable across countries.

1. The discussion could be deepened to reflect also on why there is a downward trend in service availability and correlated with other literature from the same setting.

RESPONSE

Thank you for the comment and we have not included some evidence that supports the downward trend in service availability.

2. Limitations can include the lack of any explanations for the trends observed- which would have been possible with a qualitative component to explore why some of these findings are seen.

RESPONSE

Thank you so much for the comments. Since our study included on qualitative health facility assessment, we will not be able to include qualitive information. But we have reviewed some technical reports/articles which has described

3. The service availability and readiness seem to show opposite trends - please comment and discuss

RESPONSE

Thank you and now we have some description about the service availability and readiness trend under discussion section (pp 12- 2nd paragraph).

4. Discussion on type of facility and service availability- higher facilities are expected to have more availability but primary health care strengthening is of importance in the management of chronic conditions, this requires some discussion and comment

RESPONSE
Thank you so much for the comment. We have now discussed how the primary health facilities can support the management of chronic conditions in the health care system.

Reviewer #3:

The authors report on 3 rounds of a national survey of public sector health facilities to understand the overall availability and readiness to provide care for the growing prevalence of a range of NCDs (HTN, DM, cancer and mental health). The use of the existing data is a clear strength for the manuscript, reflecting large gaps in availability and in readiness with limited improvement over time. The paper is particularly important given the growing burden of NCDs in Nigeria, the region and globally. However the paper needs revisions to be able to better communicate the results, expand on what is presented and strengthening the discussion.

RESPONSE
Thank you for the comment. We have now revised the discussion section to give more sense in communicated the finding to the wider scientific community or reader.

Introduction
The introduction is solid It would be good to understand how in the presence of rising prevalence and low system readiness, the NCD-related mortality also dropped. Also would be good to discuss in the discussion. It would also be important to frame the paper and readiness results as necessary but not sufficient for quality coverage for NCDs.

RESPONSE
Thank you so much for the comment. We have now included some description about reason behind in dropped NCD - related mortality given the rising prevalence and low health system readiness.

Methods
Was there a need to adjust for sampling since some analyses were done looking at facility type and if a national estimate is needed?

Thank you for the comment. We have taken the sample of facility from the list of health facilities as a sampling frame; we didn't follow a sampling strategy that needs adjustment/weighting. Thus, there is no need to make adjustment in analysis.

Why was 50% determined to be the threshold?

RESPONSE
Thank you for the concern. That was indicated to promote easy understating among readers.

Is there a cross walk between the areas covered and methods in the different survey types?

RESPONSE
Thank you for the good question. We would like to assure you that there was NO any methodological difference among the surveys.

Data collection-states "adapted version" of ESPA+ and SDI-which version and is there a reference?

RESPONSE
Thanks for the point raised and we have not included the reference. But the questionnaire is a standard one and has NO version. The service availability and readiness assessment (SARA) methodology was developed through a joint World Health Organization (WHO) – United States Agency for International Development (USAID) collaboration. The methodology builds upon previous and current approaches designed to assess service delivery including the service availability mapping (SAM) tool developed by WHO, and the service provision assessment (SPA) tool developed by ICF International under the USAID-funded MEASURE DHS project (monitoring and evaluation to assess and use results, demographic and health surveys) project, among others. It draws on best practices and lessons learned from the many countries that have implemented health facility assessments as well as guidelines and standards developed by WHO technical programmes and the work of the International Health Facility Assessment Network (IHFAN)

Which results were used-interview or observation?

RESPONSE
There are some data collected through observation and some though interview. This manuscript used data from the interview but there are times when we need to observe whether what we asked (could be availability of guideline, functionality of equipment and reagent) is functional or not.

Under ethical-what is meant by "sometimes health workers responsible for client services?"

RESPONSE
Thank you for the comment. We used that indicate health care workers who are providing service and available during the data collection time. This was done during data collection to avoid interviewing someone who not a primary service providing e.g health care worker is working at the administration. Actually, we have removed from the body of the revised manuscript.

Results:

The authors are very detailed in providing rates of availability and readiness, but largely repeat what is in the tables. However there are areas of details which would significantly strengthen the impact of this paper. For example, exploring where areas of gaps were across sites and going into more details of the readiness areas rather than just the mean. In addition, the table 1is hard to understand with numbers versus % and needs definitions of the facility types. The authors also note subnational differences, and so more details such as urban/rural and in the discussion why state-level differences may have been seen. This is particularly important as discussed in discussion. Statistics should be included in the results, not just the table (and should be included in the figure)-for example page 10 where it states "statistically significant"

Some analysis of overall access (availability plus readiness would also strengthen the results. Were there any tests for trends of availability and readiness? Versus T tests?

RESPONSE
Thank you for the question. We have chosen T-test because we are dealing with comparing means.

Page 8 bottom-"the sentence starting with only cancer diseases and mental illness and chronic renal diseases services were diagnosed and managed…." Is confusing and need clarification

RESPONSE
Thank you for pointing out this. We have now clarified this statement in the body of the manuscript.

Page 9: states bar graph shows trend for 3 NCDs, but then lists 4

RESPONSE
Thank you for the concern. This is because we have no data for the year 2014 and 2016, as the survey did not assess those disease entities.

Discussion
The discussion at the start is repeating results-It would be more effective to summarize broadly and then dive into the similarities and differences with previous studies. In the discussion of rising burden.

RESPONSE
Thank you for the general comment we have now revised the discussion section to be more convincing given the contradicting situation of NCD burden and health system readiness.

In limitations-would also include that private sector was not included (by my understanding). While the policy implications is a good approach-the paragraph is a little hard to follow and could be simplified for a stronger message-perhaps-rising rates of NCDs, no real change in

readiness-policy, resources need to focus. Minor-please review for sentence and grammar-such as the first sentence (readiness and readiness". The manuscript particularly the discussion would be improved by a careful review and edit

RESPONSE

Thank you for the comment. In the revised version of the manuscript, we have noted the overall aspect of the content and style of the paper and reviewed the discussion section with a maximum care.

With regards,
