# Supplementary material for: Health system’s availability and readiness of health facilities for chronic non-communicable diseases: Evidence from the Ethiopian national surveys
Source: PLoS One. 2024 Feb 23;19(2):e0297622. doi: 10.1371/journal.pone.0297622 (PMC10890768; doi:10.1371/journal.pone.0297622)
Supplement: Attachment — Submitted filename: Point_by_point response for NCD MS.docx [file pone.0297622.s003.docx]

Dear Charu C Garg (Ph.D)

Academic Editor

PLOS ONE Journal

Thanks for the comments, concerns, and revision points you and the three reviewers have provided to this manuscript titled “**Health System’s Availability and Readiness of Health Facilities for Chronic Non-Communicable Diseases: Evidence from the Ethiopian National Surveys**” number PONE-D-22-03523. We have now addressed most of the comments and amended, you could find in the file “revised manuscript track changes” and highlighted in red. And we have revised the manuscript according to the PLOS ONE style and guideline and captions are corrected.

Regarding the funding, we would like to disclose that no fund was obtained for this study and would like to write in the manuscript in the following way. “*The funders had no role in study design, data collection and analysis, decision to publish, or preparation* of the manuscript.” The authors received no specific funding for this work.”

The data availability could be described like this: - *Authors of this manuscript has not right to share or put the data on a data repository to be accessible by all who need the data. The data can be accessed through a formal request to the Ethiopian Public Health institute and it’s also available at the Website of ICF international.*

The acknowledgment section is corrected accordingly. “*We acknowledged to the Ministry of Health and the Ethiopian Public Health Institute for providing the data. As well, we are highly indebted to appreciate the contribution of study participants as well as data collectors. The surveys were undertaken by the Ethiopian Public Health Institute (EPHI), which is the research arm of the Ethiopian Ministry of Health (FMoH). They were also done in collaboration with various organizations. We would like to thank, for ICF International provided, the United States Agency for International Development (USAID), World Bank, Irish Aid, WHO and UNICEF for their technical assistance during the 2014 ESPA+. Ands the World Health Organization, World Bank and Global Fund during the 2016 and 2018 surveys*.”

Additional Editor Comments (if provided):

Thanks for submitting this paper on an important area to this journal. The paper is interesting, but can benefit from some revisions as suggested by the 3 reviewers and also my own comments in the attached document.

The methods section needs to have more clarity on defining the tracer elements. Put it in the context of WB SDI and explain the reasons for variables that were chosen as tracer values. In the tables the n values must be provided when the % are being presented for each variable.

RESPONSE:

Tracer indicators are items that can measure the specific domain of assessing service specific availability and readiness in objective information about whether or not a facility meets the required conditions to support provision of basic or specific services with a consistent level of quality and quantity. For instance, tracer items for availability and readiness of the service include trained staff, guidelines, equipment, diagnostic capacity, and medicines and commodities.

**Reviewer #1:**

The paper covers changes in health facility and readiness in managing NCDs in Ethiopia using national SARA surveys and interestingly reports a decline in many parameters. While potentially relevant for readers, it needs to address some critical issues better.
**Abstract:**

The results especially the for availability of tracer items need to be given for each disease by year for easy understanding.

RESPONSE

Thanks, you for the comment. We have now revised the abstract section considering the availability of tracer items. Some disease such as mental health and chronic renal disease have not been assessed during the 2016 and 2018. Thus, we are not able to compare with the 2014 survey result. General cancer service readiness is also not assessed during these periods rather it was specific for cervical cancer service readiness. We have described this under the limitation section.

**Main paper:**
Introduction: 40 million people were not reported to have died from NCDs but were estimated to have died. Premature deaths due to NcDs are those between 30-69 years and not below 70 years.
As the paper focuses on comparison between the three surveys, it is critical that reader knows the three survey methodology better. It will be good to tabulate details of the three surveys (sample size, sampling, tools used, other relevant measurement issues etc,).

RESPONSE

Thank you for the concern. There is NOT much methodological difference among the three surveys, but we have added more methodological details about the three surveys. This is also indicated in the Method section under Data source and Study setting and study design subsections.

The 2018 SARA (768) was conducted in the same health facilities that were included in 2016 (705). The difference is due to the facilities that the surveys were considering all hospitals to be assessed. Thus, the newly constructed and upgraded health facilities make a difference in the number. We have indicated this under the last two line of the paragraph in the Sampling and health facility sub-section.

The number and proportion of health facilities changed in the surveys in terms of primary/secondary/tertiary. Could these have affected the results and the decline is a reflection of choice of facilities than the decline. The authors have to ensure that measurement issues are not causing a bias in terms of decline of service availability and readiness. The results should also be presented by the level of facilities. Have they used a weighted prevalence to adjust for these differences?

RESPONSE

Thank you for the concern. This is not a repeated cross sectional study. The study is conducted in a representative sample of all hospitals and selected health centres and health posts. Thus, we wouldn’t expect the same health facilities to be included in different survey time. The analysis also considered the proportion which would NOT be affected by sampling since all survey considered random sampling from the list of Ethiopian health facilities as a sampling frame.

Readiness was only measured in subset of facilities that offered services. I think the denominator has to be consistent as all facilities.

RESPONSE

The denominator for service availability is all facility that is assessed in the survey, but for the readiness first that facility should provide/offer the service. That is according to the WHO Service Availably and Readiness Assessment guide.

**It is not clear how the readiness score can be that half of the facilities that offered the service had each of the input. OR was it that at least 50% of the inputs were available in a facility which had a readiness score of 50?**

RESPONSE

We have not added more explanation to make it clearer. A readiness score of 50% is on average that 50% of the health facilities assessed have all the necessary trace items to provide the service. This is just to make clearer about how the means score is calculated and interpreted.

**Table 1** - Region can be deleted. If needed a map may be provided with location of studies facilities to ensure that these are geographically well spread. As mental health and CKD were only seemed to have been assessed in 2014, these can be deleted.

RESPONSE

Thank you for the comment. We accepted the comments and have now removed the region and mental health and chronic kidney disease (Tables 1).

**Discussion** has to really focus on whether the decline is real or because of selection and measurement bias. If real, then a better explanation of the why this occurred based on the authors knowledge of context. There is no point comparing these findings with other countries as they are not very relevant.

RESPONSE

Thank you so much for the comment. We want to assure that there wasn’t any measurement bias that makes the decline in service availability and readiness, since we have used the standard survey data that have been collected in different times. We believe that all the data collection tool, measurement approach is up to the standard. In addition, we have added few contextual explanations about why the decline in service readiness.

**Reviewer #2:**

This is an important analysis in the given context and the use of standardised survey data makes it comparable across countries.
1. The discussion could be deepened to reflect also on why there is a downward trend in service availability and correlated with other literature from the same setting.

RESPONSE

Thank you for the comment and we have not included some evidence that supports the downward trend in service availability.

2. Limitations can include the lack of any explanations for the trends observed- which would have been possible with a qualitative component to explore why some of these findings are seen.

RESPONSE

Thank you so much for the comments. Since our study included on qualitative health facility assessment, we will not be able to include qualitive information. But we have reviewed some technical reports/articles which has described

3. The service availability and readiness seem to show opposite trends - please comment and discuss

RESPONSE

Thank you and now we have some description about the service availability and readiness trend under discussion section (pp 12- 2^nd^ paragraph).

4. Discussion on type of facility and service availability- higher facilities are expected to have more availability but primary health care strengthening is of importance in the management of chronic conditions, this requires some discussion and comment

RESPONSE

Thank you so much for the comment. We have now discussed how the primary health facilities can support the management of chronic conditions in the health care system.

**Reviewer #3:**

The authors report on 3 rounds of a national survey of public sector health facilities to understand the overall availability and readiness to provide care for the growing prevalence of a range of NCDs (HTN, DM, cancer and mental health). The use of the existing data is a clear strength for the manuscript, reflecting large gaps in availability and in readiness with limited improvement over time. The paper is particularly important given the growing burden of NCDs in Nigeria, the region and globally. However the paper needs revisions to be able to better communicate the results, expand on what is presented and strengthening the discussion.

RESPONSE

Thank you for the comment. We have now revised the discussion section to give more sense in communicated the finding to the wider scientific community or reader.

**Introduction**
The introduction is solid It would be good to understand how in the presence of rising prevalence and low system readiness, the NCD-related mortality also dropped. Also would be good to discuss in the discussion. It would also be important to frame the paper and readiness results as necessary but not sufficient for quality coverage for NCDs.

RESPONSE

Thank you so much for the comment. We have now included some description about reason behind in dropped NCD - related mortality given the rising prevalence and low health system readiness.

**Methods**
Was there a need to adjust for sampling since some analyses were done looking at facility type and if a national estimate is needed?

Thank you for the comment. We have taken the sample of facility from the list of health facilities as a sampling frame; we didn’t follow a sampling strategy that needs adjustment/weighting. Thus, there is no need to make adjustment in analysis.

Why was 50% determined to be the threshold?

RESPONSE

Thank you for the concern. That was indicated to promote easy understating among readers.

Is there a cross walk between the areas covered and methods in the different survey types?

RESPONSE

Thank you for the good question. We would like to assure you that there was NO any methodological difference among the surveys.

Data collection-states “adapted version” of ESPA+ and SDI-which version and is there a reference?

RESPONSE

Thanks for the point raised and we have not included the reference. But the questionnaire is a standard one and has NO version. The service availability and readiness assessment (SARA) methodology was developed through a joint World Health Organization (WHO) – United States Agency for International Development (USAID) collaboration. The methodology builds upon previous and current approaches designed to assess service delivery including the service availability mapping (SAM) tool developed by WHO, and the service provision assessment (SPA) tool developed by ICF International under the USAID-funded MEASURE DHS project (monitoring and evaluation to assess and use results, demographic and health surveys) project, among others. It draws on best practices and lessons learned from the many countries that have implemented health facility assessments as well as guidelines and standards developed by WHO technical programmes and the work of the International Health Facility Assessment Network (IHFAN)

Which results were used-interview or observation?

RESPONSE

There are some data collected through observation and some though interview. This manuscript used data from the interview but there are times when we need to observe whether what we asked (could be availability of guideline, functionality of equipment and reagent) is functional or not.

Under ethical-what is meant by “sometimes health workers responsible for client services?”

RESPONSE

Thank you for the comment. We used that indicate health care workers who are providing service and available during the data collection time. This was done during data collection to avoid interviewing someone who not a primary service providing e.g health care worker is working at the administration. Actually, we have removed from the body of the revised manuscript.

**Results:**
The authors are very detailed in providing rates of availability and readiness, but largely repeat what is in the tables. However there are areas of details which would significantly strengthen the impact of this paper. For example, exploring where areas of gaps were across sites and going into more details of the readiness areas rather than just the mean. In addition, the table 1is hard to understand with numbers versus % and needs definitions of the facility types. The authors also note subnational differences, and so more details such as urban/rural and in the discussion why state-level differences may have been seen. This is particularly important as discussed in discussion. Statistics should be included in the results, not just the table (and should be included in the figure)-for example page 10 where it states “statistically significant”

Some analysis of overall access (availability plus readiness would also strengthen the results. Were there any tests for trends of availability and readiness? Versus T tests?

RESPONSE

Thank you for the question. We have chosen T-test because we are dealing with comparing means.

Page 8 bottom-“the sentence starting with only cancer diseases and mental illness and chronic renal diseases services were diagnosed and managed….” Is confusing and need clarification

RESPONSE

Thank you for pointing out this. We have now clarified this statement in the body of the manuscript.

Page 9: states bar graph shows trend for 3 NCDs, but then lists 4

RESPONSE

Thank you for the concern. This is because we have no data for the year 2014 and 2016, as the survey did not assess those disease entities.

**Discussion**
The discussion at the start is repeating results-It would be more effective to summarize broadly and then dive into the similarities and differences with previous studies. In the discussion of rising burden.

RESPONSE

Thank you for the general comment we have now revised the discussion section to be more convincing given the contradicting situation of NCD burden and health system readiness.

**In limitations**-would also include that private sector was not included (by my understanding). While the policy implications is a good approach-the paragraph is a little hard to follow and could be simplified for a stronger message-perhaps-rising rates of NCDs, no real change in readiness-policy, resources need to focus. Minor-please review for sentence and grammar-such as the first sentence (readiness and readiness”. The manuscript particularly the discussion would be improved by a careful review and edit

RESPONSE

Thank you for the comment. In the revised version of the manuscript, we have noted the overall aspect of the content and style of the paper and reviewed the discussion section with a maximum care.

With regards,
