# Supplementary material for: Health system’s availability and readiness of health facilities for chronic non-communicable diseases: Evidence from the Ethiopian national surveys
Source: PLoS One. 2024 Feb 23;19(2):e0297622. doi: 10.1371/journal.pone.0297622 (PMC10890768; doi:10.1371/journal.pone.0297622)
Supplement: Attachment — Submitted filename: Rebuttal Letter_322023.doc [file pone.0297622.s005.doc]

**Rebuttal Letter**

Dear Charu C Garg (Ph.D)

Academic Editor

PLOS ONE Journal

Thanks for the comments, concerns, and revision points you and the three reviewers have provided to this manuscript titled “Health System’s Availability and Readiness of Health Facilities for Chronic Non-Communicable Diseases: Evidence from the Ethiopian National Surveys” number PONE-D-22-03523R1. We have now addressed most of the comments and amended, you could find in the file “revised manuscript track changes” and highlighted in red. And we have revised the manuscript according to the PLOS ONE style and guideline and captions are corrected.

Please review your reference list to ensure that it is complete and correct. If you have cited papers that have been retracted, please include the rationale for doing so in the manuscript text, or remove these references and replace them with relevant current references. Any changes to the reference list should be mentioned in the rebuttal letter that accompanies your revised manuscript. If you need to cite a retracted article, indicate the article’s retracted status in the References list and also include a citation and full reference for the retraction notice.

Additional Editor Comments (if provided):

While the paper has been greatly improved, I find that all the changes made in the track changes version, did not show up in the clean version. Please upload correct versions. I think that the reviewer that has made comments specifically about sampling etc. did not see the track change mode but the clean version, where those changes are missed.

1. Agree with the reviewer that results in abstract are very descriptive with just the numbers. Can we highlight the major findings under the results? The % may only be presented for 2018 and show where there are improvements from previous years and why? Also focus on the types of facility and regional differences to bring about the difference in performance.

Response: It is a good comment suggested regarding the numbers depicted in the result section of the abstract. We present the finding in the form with types of facilities, residence, and region as described in Table 1. However, we are not compared and analyzed to find out the differentials for both availability and readiness across different facilities, region and residence, and this is not the scope of the paper we are addressed.

2. Number of facilities is not important in the methods in abstract. Can you briefly say some tracer elements, especially which are significant in your study and also discuss these under results in the abstract?

Response: It is a good comment and revised in the current file.

3. Needs editing – e.g. Ethiopia repeated twice in abstract under conclusions; Para 1 in introduction;

Response: It is a good comment and revised in the current file.

4. It is mentioned that the analysis was done across types of facilities, residence, region – the differentials for both availability and readiness across different facilities should be more clearly highlighted. There is only one small discussion about training in the discussion across the facilities.

Response: It is a good comment and revised in the current file. We present the finding in the form with types of facilities, residence, and region as described in Table 1. However, we are not compared and analyzed to find out the differentials for both availability and readiness across different facilities, region and residence, and this is not the scope of the paper we are addressed.

5. I feel discussion needs to be rearranged. Not sure about the significance of giving prevalence in beginning of the 2nd para of discussion. That needs to be placed in a context. As also mentioned by one of the reviewers it would be useful to critically discuss the results and state why availability or readiness is low and when it improved, why and if not, why not. You could also consider organizing the discussion around availability and readiness or by diseases. Under each of these clearly organize which tracer elements are strengthened. And at what level of facility. Too much of repetition after every sentence about Uganda, Ghana and Zambia, can you first discuss your major findings and then say what these other studies say about your results. Might make it easier to follow.

Response: We have organized the paper as per the reviewers and editor feedback to improve the quality of the paper. We removed the prevalence aspect in the discussion section.

Reviewers' comments:

Reviewer's Responses to Questions

Comments to the Author

1. If the authors have adequately addressed your comments raised in a previous round of review and you feel that this manuscript is now acceptable for publication, you may indicate that here to bypass the “Comments to the Author” section, enter your conflict of interest statement in the “Confidential to Editor” section, and submit your "Accept" recommendation.

Reviewer #1: All comments have been addressed

Reviewer #2: All comments have been addressed

Reviewer #3: (No Response)

Response: It is a good comment and appreciates the reviewers thought to improve the manuscript.

2. Is the manuscript technically sounds, and do the data support the conclusions?

The manuscript must describe a technically sound piece of scientific research with data that supports the conclusions. Experiments must have been conducted rigorously, with appropriate controls, replication, and sample sizes. The conclusions must be drawn appropriately based on the data presented.

Reviewer #1: Yes

Reviewer #2: Yes

Reviewer #3: Partly

Response: Thank you for your comment.

3. Has the statistical analysis been performed appropriately and rigorously?

Reviewer #1: Yes

Reviewer #2: Yes

Reviewer #3: I Don't Know

Response: Thank you for your comment and do acknowledge the feedback.

4. Have the authors made all data underlying the findings in their manuscript fully available?

The PLOS Data policy requires authors to make all data underlying the findings described in their manuscript fully available without restriction, with rare exception (please refer to the Data Availability Statement in the manuscript PDF file). The data should be provided as part of the manuscript or its supporting information, or deposited to a public repository. For example, in addition to summary statistics, the data points behind means, medians and variance measures should be available. If there are restrictions on publicly sharing data—e.g. participant privacy or use of data from a third party—those must be specified.

Reviewer #1: Yes

Reviewer #2: Yes

Reviewer #3: Yes

Response: Thank you for your feedback.

5. Is the manuscript presented in an intelligible fashion and written in standard English?

PLOS ONE does not copyedit accepted manuscripts, so the language in submitted articles must be clear, correct, and unambiguous. Any typographical or grammatical errors should be corrected at revision, so please note any specific errors here.

Reviewer #1: Yes

Reviewer #2: Yes

Reviewer #3: No

Response: Thank you for your comment and do acknowledge the feedback.

6. Review Comments to the Author

Please use the space provided to explain your answers to the questions above. You may also include additional comments for the author, including concerns about dual publication, research ethics, or publication ethics. (Please upload your review as an attachment if it exceeds 20,000 characters)

Reviewer #1: (No Response)

Reviewer #2: (No Response)

Reviewer #3: Review PLOS

The authors have leveraged existing facility assessment data to highlight the lack of progress (or even decline) in facility readiness to treat the rising burden of NCDs in Ethiopia. The analysis to increase the use of these data is to be applauded, but the paper as written needs strengthening to better convey and discuss the findings. I have included some broader areas as well as some details where edits are needed which would make the paper stronger (as well as a review for grammar-long sentences, a page and ½ paragraph in discussion etc.)

Response: Thank you for your comment and do acknowledge the feedback.

1. Abstract

a. In the abstract, only listing the numbers makes it hard to read in the medication readiness. Reorganizing to show persistently low by area would be better, although some may have improved albeit remaining low For example levels of medications remained low between 2014 and 2018 for Diabetes (____ to ___%), cardiovascular disease (____ to ____) etc.

Response: Thank you for your comment and do acknowledged the feedback.

b. Were there any statistics done to see of these were insignificant changes or if some actually increase

Response: We use Fisher's exact test was used to assess the difference in the mean availability of tracer items as we see in Table 3.

2. Introduction

a. The statement about NCD deaths in Ethiopia needs a reference

Response: Thank you for your comment and amended accordingly.

b. I am also confused by the sentence” The Disability Adjusted Life Years (DALYs) increased from below 20% in 1990 to 69% in 2015 and the incidence and prevalence of NCDs are become rising”

Response: Thank you for your comment. Both the prevalence and incidence of NCDs getting higher in the country. The evidence generated from Global Burden of Disease indicates this circumstance in more detailed manner (i.e. https://pubmed.ncbi.nlm.nih.gov/28732542/, https://www.ajol.info/index.php/ejhd/article/view/178817)

c. The first paragraph gets a bit repetitive and hard to follow esp. as all about increase but the final sentence states “The death rates of NCDs causes declined by 40% from 1990 to 2015 and also from the 680.9 per 100,000 crude all-cause mortality rate, 286.9 (95% UI: 188.1–423.0) per 100,000 people” which is confusing.

Response: Thank you for your comment and the statement is now clear in the amended manuscript accordingly.

d. The sentence which is long on other studies “These studies, however, are limited as they focus on a particular or selected set of NCDs 18-20.Other studies in the country also showed that from a total 21 public health facilities surveyed, only 38.1% had all the categories of health professionals as compared to the national standards and 85.2%, of the facilities fulfilled the criteria for basic equipment and 47.7% of the facilities did not fulfill the criteria for infection prevention supplies21 and this is linked with the availability of essential equipment and drugs, health workers' communication, health care provided, and attitude of health workers had positive association with client satisfaction with various health programs in the health system” is hard to understand-is this about NCDs

Response: It is a good comment and we removed the statement from the body of the manuscript.

e. The last sentence highlights other gaps than those covered in the study-so would be good to explain which of the gaps this study described

Response: The gap identified in other studies is basically focused on neither specific disease entity nor items. So, our study conducted in a comprehensive manner addressing a wide range from the availability of guideline and essential drugs, training, logistic and equipment.

3. Methods

a. How did the surveys in 2014 versus 2016 and 18 differ (if at all). Were the sampling different (a little hard to tell)

Response: It is a good comment and there is no difference among the surveys methodology that differ for the interpretation of the finding. However, we described the approach and methods used in more detailed manner in the method section of the manuscript. To be more elaborative, in 2014 sampling,

all hospitals were included and a representative sample of Health Centers, Health posts and Clinics were included. While, in 2016 sampling, all hospitals were included and a representative sample of Health Centers, Health posts and Clinics were included and in 2018 sampling, all hospitals were included and a representative sample of Health Centers, Health posts and Clinics were included. These imply, no sampling difference in the surveys.

b. Were private facilities included (if not need to be clear this is only about public facilities?)

Response: We have included private facilities and described in Sampling of health facilities part in method section. In addition, in both SPA 2014, SARA 2016 and SARA 2018 the managing authority for the Clinics are private.

c. What language was used ? If translated how was that done?

Response: In SPA 2014, the standard English questionnaire was translated to the three local languages: Amharic, Afaan Oromo and Tigrigna while in SARA 2016, the standard English questionnaire was translated to Amharic and lastly in SARA 2018, the standard English questionnaire was translated to Amharic.

d. What does the references for “The availability of the services and readiness of health facilities for diabetes, cardiovascular, and chronic respiratory diseases were investigated in the ESPA+ 2014, and the two recent (2016 and 2018) SARA surveys”- was this referencing for the definitions. I would also think that referral does not constitute availability. Would consider a sub analysis removing that option. Also this is not consistent with what is stated in the following section “We defined facility ‘'availability" as the percentage of facilities in the sample that said they offered the service in question, which considered whether the diagnosis and/or management services were available. The results section is also confusing as seems like a broader set of requirements for availability.

Response: The scope of the study and the data used is coming from a national survey conducted using WHO standard approach used widely in the globe for assessing availability and readiness of service provision. All in all, the method and definitions are also following the same standard procedures.

it refers as a definition for “availability of services for non-communicable diseases”. And in the sentence “The service was considered to be available if a facility manages, diagnoses, treats or refers a patient coming to the facility for common NCDs.” The word refers inserted by mistake. It needs to be replaced by “prescribe”

e. The description of calculation of readiness was a little hard to follow-is readiness a dichotomous outcome and so as stated 50% means 50% are ready but the following statement notes” A readiness score of 50 signifies that, on average, half of the facilities that offered the service had each of the requisite inputs for delivering that service. The calculated indices were compared to an agreed cutoff point of 50%. A facility index that was below 50% was considered as `not ready' to manage NCDs.” Can this be clarified

Response: Readiness score is not a dichotomous outcome. For instance, in SARA 2016, related to “Cervical cancer”, 53% of facilities had guideline, 61% of facilities had at least 1 trained staff, 96% of facilities had speculum, 77% of facilities had acetic acid (These are required inputs/items to provide cervical cancer services). It implies the mean availability of these 4 tracer items readiness score will be ([53%+61%+96%+77%]/4)=72%. It reveals this facility was ready to provide cervical cancer service (>50%).

f. How were missing data handled if any)

Response: If there is missing data, it was excluded from analysis

4. Results

a. Table 1 needs some work-it says % but some are >100%

i. Some of the facility types are not described and if health posts are not included-which would these be?

Response: the health posts are removed from this study as they did not provide the services and already described in the method section under statistical analysis.

b. Were there any differences in facilities studied between the years? As the number dropped

Response: of course, the sample we use from the list of health facilities varies across the year.

c. How were the p values determined (table 3) and seems like these should be referenced in statements are made about decrease (or not) and if and how adjusting for facility type (ex for differences based on rural/urban). For Table.3 I assume this is absolute change (but needs description in methods and table). Page 10 also state the p value not just say significant

Response: it is a good point and we have amended that in the method section.

d. Given the range in the different components of the readiness, a discussion of where the areas that remain low versus those which are stronger may help

Response: it is a good comment and updated accordingly.

e. Some information should be first presented /reported in results beyond the table versus discussion -such as difference in availability in higher level facilities-would consider decreasing words in methods and expand results. Same for sector differences if any existed

Response: there is no analysis carried out to compare health facilities in which it is not the aim of the study. However, the authors believe from experience that the hospitals provide more service for NCD in terms of availability and readiness to provide the intended services than health centers.

f. Table 2:

i. Is the mean the average of the average of total facility score?

Response: it is a good point and it is not like that. The mean availability of tracer items for each disease that are described in the table and their tracer element is the one to be measured.

5. Discussion

a. First sentence needs editing and in the first paragraph would not just report the details, but have a broader summary-where were areas of particular weakness within and across NCDs for example. Similar comments are throughout the discussion. Some of this is found in the overly long paragraph (page 11-12) but should be a separate paragraph better explained

Response: the first paragraph outlined the summery of the main finding. But, we tried to shorten the paragraphs accordingly in the revised version of the manuscript.

b. It is curious that readiness for DM is highest but availability is lowest. In the discussion this should be discussed and what this means in terms of accessibility (which facilities had availability and hence were measured for readiness-more urban? Higher level?

Response: approximately the service availability and readiness correspond for the service for diabetes mellites. However, the service availability and readiness for DM is higher in urban and facility type where most of the service is higher for availability and readiness in hospital than PHC setting in the country.

c. Would consider a measure of access-which would what % of all facilities, had the care available and adequate readiness-I suspect the % would be even lower

Response: It is out of the scope of the methodology for assessing the health facilities in terms of accessibility.

d. The PEN standards are important, but in bringing them in, a brief description of if and how they differ from the standards used in the study are needed

Response: It is a good point. However, the PEN standard also follow the same approach that the SARA and SPA measurement used as a modality of checking the and strengthen the health services delivery and the management of the PHC system for diagnosis and treatment of selected NCDs.

e. For limitations-what does “seemed” mean for oversampled?

Response: We have removed the wording, it is typo error.

f. In the last paragraph before conclusions-the first sentence is a it repetitive. Also, is the problem policy or funding of the policy or funding but supply and training? I did not see that discussed. Th conclusion is also. A bit repetitive and some recommendations are perhaps not as strongly supported (ex. Training-is this a response to trained HCWs-or is the gap in actual number of HCWs-the deficits in materials also seems to be the most important bottleneck?

Response: It is a good point. In the conclusion we are summarizing the few bullet points of what is actual conclude from this study. The training is to support the HCW technical capacity in managing the patients. As suggested from the reviewer, logistic and materials availability for treating cases is also important and addressed in the revised version of the manuscript.

7. PLOS authors have the option to publish the peer review history of their article (what does this mean?). If published, this will include your full peer review and any attached files. If you choose “no”, your identity will remain anonymous but your review may still be made public.

Do you want your identity to be public for this peer review? For information about this choice, including consent withdrawal, please see our Privacy Policy.

Reviewer #1: Yes: Anand Krishnan

Reviewer #2: No

Reviewer #3: No

Best regards,
