# Supplementary material for: Health system’s availability and readiness of health facilities for chronic non-communicable diseases: Evidence from the Ethiopian national surveys
Source: PLoS One. 2024 Feb 23;19(2):e0297622. doi: 10.1371/journal.pone.0297622 (PMC10890768; doi:10.1371/journal.pone.0297622)
Supplement: Attachment — Submitted filename: Rebuttal Letter 4_10142023.docx [file pone.0297622.s006.docx]

**Rebuttal Letter 4**

Dear Charu C Garg (Ph.D)

Academic Editor

PLOS ONE Journal

Thanks for the comments, concerns, and revision points you and the three reviewers have provided to this manuscript titled “Health System’s Availability and Readiness of Health Facilities for Chronic Non-Communicable Diseases: Evidence from the Ethiopian National Surveys” number PONE-D-22-03523R1. We have now addressed most of the comments and amended, you could find in the file “revised manuscript track changes” and highlighted in red. And we have revised the manuscript according to the PLOS ONE style and guideline and captions are corrected.

Journal Requirements:

Please review your reference list to ensure that it is complete and correct. If you have cited papers that have been retracted, please include the rationale for doing so in the manuscript text, or remove these references and replace them with relevant current references. Any changes to the reference list should be mentioned in the rebuttal letter that accompanies your revised manuscript. If you need to cite a retracted article, indicate the article’s retracted status in the References list and also include a citation and full reference for the retraction notice.

Additional Editor Comments:

he paper has been substantially improved. There are minor comments especially related to table formats, grammatical errors and a few explanations. Once those are addressed, the paper can be accepted,

[Note: HTML markup is below. Please do not edit.]

Reviewers' comments:

Reviewer's Responses to Questions

**Comments to the Author**

**Question** 1. If the authors have adequately addressed your comments raised in a previous round of review and you feel that this manuscript is now acceptable for publication, you may indicate that here to bypass the “Comments to the Author” section, enter your conflict of interest statement in the “Confidential to Editor” section, and submit your "Accept" recommendation.

Reviewer #3: (No Response)

Reviewer #4: (No Response)

**Response**: It is a good reflection and thank you for the feedback.

**Question** 2. Is the manuscript technically sound, and do the data support the conclusions?

The manuscript must describe a technically sound piece of scientific research with data that supports the conclusions. Experiments must have been conducted rigorously, with appropriate controls, replication, and sample sizes. The conclusions must be drawn appropriately based on the data presented.

Reviewer #3: Yes

Reviewer #4: (No Response)

**Response**: Thank you for the feedback and for the positive impression.

**Question** 3. Has the statistical analysis been performed appropriately and rigorously?

Reviewer #3: Yes

Reviewer #4: I Don't Know

**Response**: Thank you for the feedback and for the positive impression.

**Question** 4. Have the authors made all data underlying the findings in their manuscript fully available?

The [PLOS Data policy](http://www.plosone.org/static/policies.action#sharing) requires authors to make all data underlying the findings described in their manuscript fully available without restriction, with rare exception (please refer to the Data Availability Statement in the manuscript PDF file). The data should be provided as part of the manuscript or its supporting information, or deposited to a public repository. For example, in addition to summary statistics, the data points behind means, medians and variance measures should be available. If there are restrictions on publicly sharing data—e.g. participant privacy or use of data from a third party—those must be specified.

Reviewer #3: Yes

Reviewer #4: (No Response)

**Response**: Thank you for the feedback and for the positive impression.

**Question** 5. Is the manuscript presented in an intelligible fashion and written in standard English?

PLOS ONE does not copyedit accepted manuscripts, so the language in submitted articles must be clear, correct, and unambiguous. Any typographical or grammatical errors should be corrected at revision, so please note any specific errors here.

Reviewer #3: No

Reviewer #4: Yes

**Response**: Thank you for your comment and do acknowledge the feedback.

6. Review Comments to the Author

Please use the space provided to explain your answers to the questions above. You may also include additional comments for the author, including concerns about dual publication, research ethics, or publication ethics. (Please upload your review as an attachment if it exceeds 20,000 characters)

Reviewer #3: The authors have worked to respond to the comments. The paper still requires a careful read for typos and grammar. For example, in the abstract, “Proposition of facilities that
offered the service for diabetes, cardiovascular disease, chronic respiratory disease, cancer
diseases, mental illness, and chronic renal diseases was calculated to measure health service
availability” should be corrected to proportion. In the introduction “and the incidence and prevalence of NCDs are become rising”

**Response**: Thank you for your comment and do acknowledge the feedback.

**Question** :Abstract: I was not sure what “However, in 2014 , the mean availability of
tracer items was 7% each for mental illness and chronic renal diseases, each respectively” signified-what were the results in more recent years? Same comment for discussion

**Response**: It is a good point raised by the reviewer. It is to mean , “ However, in 2014 survey year” , we added the word survey year so that it will suggest the data collection period where the survey has been conducted.

**Question**: Methods: I remain confused about “A readiness score
of 50 signifies that, on average, half of the facilities that offered the service had each of the
requisite inputs for delivering that service. The calculated indices were compared to an agreed
cutoff point of 50%. A facility index that was below 50% was considered as `not ready' to
manage NCDs.”. This seems to imply use overall and then later in the sentence about an individual facility. There for is a score of 40% that 40% of facilities were above 50%??
**Response**: It is quite good point suggested by the reviewer. Actually in the method section of “ measurement of variables” , it is clearly mentioned that the readiness score was calculated with the following domains: included trained staff, guideline, equipment/supplies; medicines and commodities, and diagnostics capacity. So the cut of point of 50% will be surfaced to assess the health facility capacity in that line to differentiate the health facility readiness for managing NCDs.

**Question**: Results- Statistically significant is not adequate-you need to put in the results in the text

**Response**: it is a good comment forwarded by the reviewer and amended in the revised file.

**Question**: The number of % of facility by type seems to have changed a lot across the surveys-with many more lower level facilities early on. How did the authors adjust and if not, need to add to limitations that some change could be due to different make-up of the sample.

**Response**: the distribution of facilities by type, some of the heath facilities types are overrepresented and some types are underrepresented. For example, primary hospitals in Ethiopia account for only about 1% of all health facilities. Most of the country health facilities are designed under primary health care(PHC) where the majority of the health facilities were run by the heath center and health post (i.e. In actuality, about 65% of health facilities in Ethiopia are health posts).

**Question**: In the discussion, please include the dates for the surveys being used in comparisons (South Africa and Uganda). Same for the data from Ethiopia

**Response**: It is a good comment and amended in the revised manuscript

**Question**: Table 1: Would be easier to interpret if the columns included both % and n rather than having the reader calculate the number of facilities

**Response**: It is a good comment. Considering the numbers of health facilities of each type and national and regional levels in to account for the weighted/ adjustment , so that the distribution of facilities by type’s contribution to the total is proportionate to reflect the actual distribution or total number of health facilities in Ethiopia. Hence, the weighted percent is the one depicted in the table , that actual show the true picture of the health facilities in the country. The explanation is the percentages in the table are weighed and the number of facilities (n) in the last columns should be Unweighted. Unfortunately, we have included the weighted for SPA+ 2014. Now we have changed to Unweighted numbers (n= 873). In principle we can say/write the x %age of facilities offer diabetes service in 2014 in text but we can't put the observation (n) for every %ages in the table, It's conventional that, we need to weight estimates or percentages to make sampling adjustment for disproportional sampling and non-response rate at the facility type and regional level. This is a guiding principle for the analysis of SPA data.

**Question**: Table 3: Would limit p values to 2 decimal points if not significant. I also assume difference is absolute, nor relative but that should be made clear

**Response**: we have rounded up to two decimal points for the p-value and it is an absolute difference.

**Question**: Reviewer #4: Substantial revisions have been made from the feedback of previous reviewers. I wish to however draw your retention to table 1. In the last row of totals, it is not clear what it represents. Attempt to add urban and rural located facilities gives conflicting totals from what is imputed in most of the cells. It may also be important to give a bit more details about the table as a footnote, including spelling out abbreviations/acronyms so that the table can be understandable if standing alone (this should also apply to other tables/figures). it may also be important to include the denominator number for each characteristic being measured for a better understanding of the proportions.

**Response**: It is a greet feedback that posed from the reviewers point of view. We will improve the design and layout as per the recommendation from the reviewer side.
